# Production, Biodegradation, and Recalcitrance of Organic Carbon from Cultivated *Saccharina latissima* in Norway

**DOI:** 10.1021/acs.est.6c05450

**Published:** 2026-08-11

**Authors:** Luiza Neves, Sebastian Aker, David Aldridge, Deni Ribičić, Anders Brunsvik, Ole Jacob Broch, Jorunn Skjermo, Murat Van Ardelan

**Affiliations:** † Department of Chemistry and Biomedical Science, 8018 Norwegian University of Science and Technology NTNU, NO-7491 Trondheim, Norway; ‡ Fisheries and New Biomarine Industries, 555971 SINTEF Ocean AS, Sealab, 7010 Trondheim, Norway; § Department of Biology, 8018 Norwegian University of Science and Technology NTNU, NO-7491 Trondheim, Norway; ∥ Aquaculture, SINTEF Ocean AS, Sealab, 7010 Trondheim, Norway; ⊥ Biotechnology and Nanomedicine, SINTEF Industry AS, NO-7465 Trondheim, Norway

**Keywords:** DOC, RDOC, Cultivation, Sequestration, Sugar kelp, mCDR, MRV, Mariculture

## Abstract

Particulate (POC) and dissolved (DOC) organic carbon released during macroalgae cultivation has been shown to lead to carbon burial and export to the open ocean and deep sea. The recalcitrant portion of the DOC (RDOC) is a critical contributor to carbon sequestration and a major knowledge gap. We investigate the production and bioavailability of DOC released from *Saccharina latissima*, a primary cultivar in Europe, with a focus on RDOC quantification. DOC release rates were 0.5–1.4 mg C g­(dw)^−1^ h^–1^ within 24 h, and concentrations stabilized after 187 days of biodegradation, at 37–79% (mean 58%) from initial levels. The kelp released 920 (17%) unique compounds, including large quantities of sulfur-containing molecules (CHOS, CHONS). Increasing recalcitrance was observed in kelp and control treatments, despite small variations in CRAM molecules. Microbial biodegradation generated 1,805 (34%) new molecules, including 1,207 in the recalcitrant DOM fraction. Island of Stability (IOS) analysis revealed an intensity-weighted abundance of 0.2% RDOC in kelp treatments, with persistence on geological time scales. Beyond its biomass applications, macroalgae cultivation enables scalable CO_2_ sequestration in the oceans through RDOC production from continuous release and degradation of DOC and POC during grow-out, in itself a value-added product.

## Graphical abstract

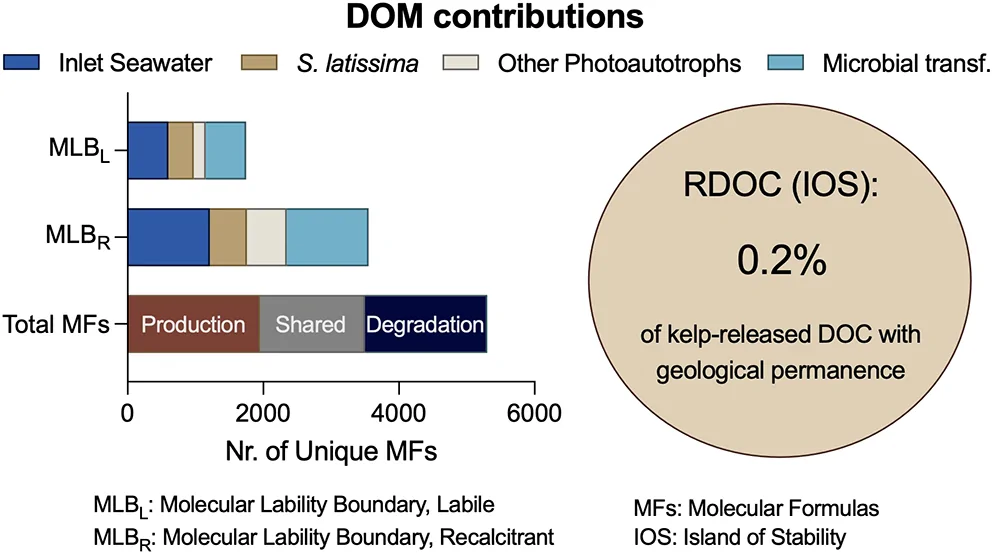

## 1 Introduction

Marine dissolved organic carbon (DOC) is the largest reservoir of reduced carbon on Earth, around 660 Pg C, almost as much carbon as in the atmosphere itself.^1^ By contrast, the particulate organic carbon (POC) pool constitutes approximately 2 to 20 Pg C, including all living biomass from the upper euphotic layer to about a 200 m depth.^2,3^ Due to the flux of CO_2_ through the ocean-atmosphere interface, the oceans have taken up around 25% of the anthropogenic carbon released since the mid-19th century.^4^ Removal of CO_2_ from the ocean may, therefore, lower atmospheric CO_2_ concentrations. Cultivation of macroalgae or restoration of kelp forests have been heralded as two biologically based/nature-based solutions for marine carbon dioxide removal (mCDR) from the ocean.^5–8^ The goal of such mCDR initiatives is either to increase the primary production and the vertical flux of carbon in the ocean (the biological carbon pump) or to increase primary production for harvest and stabilize the carbon in such a way that it is not immediately degraded and released to the atmosphere-ocean system (e.g., production of biochar).

Macroalgae are known important sources of DOC in coastal waters and of allochthonous carbon in the open ocean and deep sea,^9–15^ yet considerable knowledge gaps remain regarding DOC seasonal production, molecular composition, bioavailability, and transformation into RDOC for most macroalgae species.^13,16–18^ Labile DOC (LDOC) is the dissolved organic carbon that is readily bioavailable and rapidly consumed by microbes, including lipid-, peptide-, amino-sugar-, and carbohydrate-like compounds.^19^ Recalcitrant DOC (RDOC) has longer residence times in seawater due to the buildup of humic-like compounds, phenols, and polycyclic aromatic compounds which are less bioavailable for breakdown.^20–22^ Microorganisms play a fundamental role in the consumption and transformation of marine DOM, altering their chemical composition and bioavailability.^23,24^ RDOC, which constitutes over 90% of global ocean DOC and is a long-term buffer in the global carbon cycle,^16^ may also include diverse labile compounds in such low concentrations that they remain below the uptake thresholds by microbes.^25,26^

Long-term incubation experiments are often used to study the evolution of recalcitrant DOM, with time windows used as indicators of bioavailability. These hypothesize that labile DOC is mostly consumed within the first 30–60 days of microbial degradation, while recalcitrant DOC is that which is left after stabilization of DOC concentrations around 150 days,^15,17,27^ although Chen et al. state that this stabilization may take over 6 months.^28^ However, stabilization in concentrations does not preclude stabilization in DOM composition to inert molecular forms, and as laboratory studies are inherently time-limited, long-term permanence in DOC/DOM remains difficult to quantify. Fourier transform ion cyclotron resonance mass spectrometry (FT-ICR MS) is a state-of-the-art technique for analyzing DOM recalcitrance, allowing thousands of individual molecules to be identified from complex organic mixtures in DOM more accurately than any other mass spectrometry technique.^20,29–31^ Very few studies have used FT-ICR MS to study the composition of DOC produced from cultivated kelp, for instance, with *Saccharina japonica* ^17,27,32^ and *Ulva* ^28^ in China and Japan. Yet, the DOM composition and recalcitrance of *Saccharina latissima* have yet to be documented.

In this study, a two-part incubation experiment is used to measure production and biodegradation of organic carbon from cultivated *S. latissima*, paired with analysis of bacterial community composition, DOM composition and recalcitrance. Unique molecular species contributions from the kelp to bulk DOM are discerned, relative to other sources, for both labile and recalcitrant fractions. Analysis of CRAM molecules and the island of stability (IOS) is performed to further assess compounds with long-term permanence.

## 2 Materials and Methods

### 2.1 DOC Production Experiment and Sampling

Three sets of 12 20 L incubation containers were prepared, consisting of light and dark treatments for kelp and seawater controls (Figure 1). All containers (Collapsible Polyethylene Containers, Cole-Palmer, USA) were acid washed with 1 M HCl and then rinsed with distilled water. Natural seawater from an 80 m depth in Trondheim fjord (average 10.6 °C and 34.4 salinity) was filtered through a 200 μm plankton mesh to remove larger phytoplankton, zooplankton, and any debris, before filling containers. Cultivated *S. latissima* deployed in January 2022 was collected in May from the cultivation site in Skarvøya, Hitra (63° 39′0″ N, 8° 38′57″ E) and kept in an acclimatization tank at 10 °C overnight. All kelp was similar in shape and size (70 ± 7 cm length), with negligible bryozoan biofouling. Incubators were submerged 2 m (similar to the sea cultivation depth) over 3 days inside a basin which provided daily average seawater temperatures of 10.4–11.7 °C, natural daylight, and daily average surface light intensity (E_PAR_) of 21.1–36.0 mol m^–2^ d^–1^ (Figure S1). Inlet seawater and an acclimatization tank were sampled at T_0_, and one set of 12 incubation containers was sampled after 5, 24, and 48 h. An additional 6 containers (3 kelp, 3 controls) were sampled after 72 h and then used to set up the biodegradation study (section 2.2). At every sampling point, temperature, dissolved oxygen, and pH were measured upon opening the containers, and the kelp was swabbed for bacterial community analysis. Seawater samples were taken for analysis of POC and DOC concentrations, dissolved inorganic carbon (DIC), total alkalinity (TA), bacterial communities, and DOM characterization, as described below.

**Figure 1:**
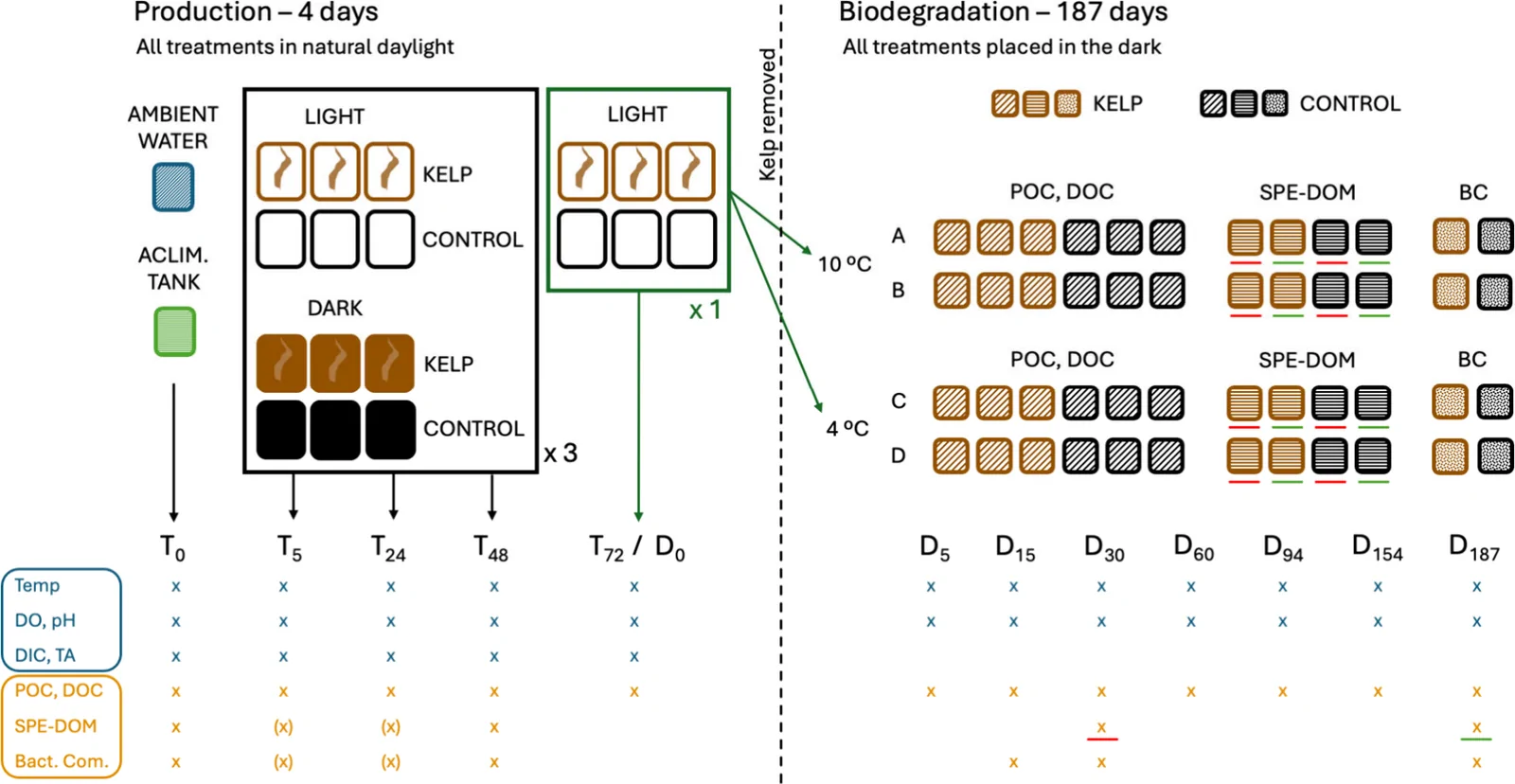
Incubation experiment setup, showing the number of treatments, replicates, and sampling times in hours for the production experiment and in days for the biodegradation experiment. (x) are samples taken but not analyzed.

#### 2.1.1 Sampling Bacterial Communities from Kelp Surface and Seawater

Microbiota were collected from the up-facing kelp surface using a swab in a meristem-to-tip direction covering both mid and edge tissues and then preserved in DNA/RNA Shield (ZymoByomics, CA, USA). For seawater bacterial samples, 1 L from each incubation container was filtered through a 47 mm, 0.22 μm Durapore PVDF membrane filter and stored in 2 mL Eppendorf biopur vials. All samples were kept at −20 °C and in darkness until processing. Here, the DNA was extracted using a DNA MiniPrep kit (ZymoBIomics, CA, USA) according to manufacturer’s instructions. DNA was eluted in DNase/RNase-free water and quality assessed using NanoDrop (Thermo Fisher Scientific, USA) and Qubit 2.0 (Thermo Fisher Scientific, USA). Sequencing was conducted by a commercial provider on a DNBSEQ platform (BGI Tech Solutions, Hong Kong, China), on previously generated amplicons using a 338F (5′- ACTCCTACGGGAGGCAGCAG-3′) and 806R (5′-GGACTACHVGGGTWTCTAAT-3′) primer set covering the V3–V4 hypervariable region of the 16S rRNA gene utilizing 300 bp paired-end chemistry.

#### 2.1.2 DOC and POC Concentrations

For each incubation treatment and replica, 1 L of seawater was filtered through precombusted, 47 mm, 0.7 μm GF/F Whatman filters in vacuum filtration systems connected to a water vacuum pump. DOC samples were collected in triplicate into precombusted (450 °C, 8 h) and prerinsed 40 mL glass vials and immediately acidified with 6 M HCl to pH < 2. All samples were kept at −20 °C and in darkness until analysis. The GF/F filters were analyzed for POC at SINTEF Ocean, Trondheim, Norway, and DOC was analyzed with a TOC-L Shimadzu analyzer at GEOMAR Helmholtz Centre for Ocean Research, Kiel, Germany. Hansell Consensus Reference Material (CRM) from the University of Miami, USA was used to verify accuracy. Vial caps were washed in 1 M HCl for 2 h, followed by soaking in methanol for 8 h, and then drying at 60 °C for 1–2 h. All filtration equipment was thoroughly rinsed between replicates and treatments with Milli-Q water and acid washed between experiment days.

#### 2.1.3 DIC and Total Alkalinity

250 mL Duran glass bottles were filled almost to the top (ca. 270 mL) using flexible silicone tubing to siphon the sample in a “CO_2_ clean” way, with as few bubbles and turbulence as possible, leaving approximately 1% of total volume as headspace. Samples were preserved with 100 μL of saturated HgCl_2_, sealed, kept cool and dark, and shipped immediately after collection for analysis at NIVA Institute, Oslo, Norway.

#### 2.1.4 DOM Extraction for Molecular Characterization

Two L of seawater from each incubation container, the inlet seawater, and three Milli-Q water blanks was used for molecular characterization with FT-ICR MS. Samples were filtered through precombusted (450 °C, 8 h) 47 mm, Whatman GF/F filters, exchanging filters as needed to prevent clogging and then acidified with 6 M HCl to pH < 2 to prevent further bacterial degradation. Prior to characterization, DOM samples must be concentrated and desalted.^33^ DOM was isolated by solid-phase extraction (SPE) according to Dittmar et al.^34^ using PPL cartridges (Agilent Bond Elut, 500 mg), widely used for their ease of operation and relatively high DOC recoveries in seawater, 40–60%. Cartridges were presoaked in methanol for 2 h before extraction to enhance solvent permeation during rinsing of the sorbent.^35^ Once filtered and acidified, seawater samples were loaded onto the SPE-PPL sorbents, with the flow rate not exceeding 30 mL/min. In the washing step, 0.1% formic acid was used instead of HCl to reduce interference from chloride ions in downstream analysis.^35^ Final elution with LCMS-grade methanol slowly gathered 6 mL of concentrated solution into 20 mL precombusted (450 °C, 8 h) glass vials, which were kept at −20 °C and in darkness until analysis at SINTEF Industry, Trondheim, Norway.

### 2.2 DOC Biodegradation

The last 6 incubation containers of the production experiment were sampled and redivided for the biodegradation experiment (Figure 1). 5% of the sample volume was filtered through precombusted 1.2 μm GF/C filters allowing passage of autotrophic and heterotrophic microorganisms < 1.2 μm, serving as an inoculum^36^ for samples in the new setup. Autotrophic growth was assumed to be prevented due to storage in darkness. The remaining seawater was filtered with precombusted 47 mm, 0.7 μm GF/F Whatman filters, and the inoculum was added. New treatments were kept at 4 and 10 °C, representing temperatures in the water column, and sampled after 0, 5, 15, 30, 60, 94, 154, and 187 days, based on reported labile and recalcitrant windows from similar studies.^13,17,27,32,37^ On every sampling date, containers were shaken vigorously allowing more heterogeneity in the sample, and temperature, dissolved oxygen, and pH were measured immediately upon opening. 250 mL per replica and treatment was filtered for analysis of POC and DOC concentrations (cf. section 2.1.2). After 30 and 187 days, 2 L samples were taken for DOM characterization (cf. 2.1.4), and 0.5 L samples were taken for bacterial community analysis after 15, 30, and 187 days (cf. 2.1.1).

### 2.3 DOM Molecular Composition and Recalcitrance

DOM was analyzed by FT-ICR MS with electrospray ionization (ESI) in negative ion mode and a mass accuracy of 0.1 ppm. Peaks were considered with a signal-to-noise ratio greater than 3 (*S*/*N* > 3) and a mass range of *m*/*z* 200–800. Prior to analysis the FT-ICR MS instrument was calibrated with a 0.1 mg/mL solution of arginine (50/50 acetonitrile water) at a flow of 10 μL/min to verify signal intensity and accurate mass calibration. Molecular formulas (MFs) containing C, H, O, N, S, and/or P were assigned to mass spectral peaks according to published rules^38,39^ and limited a posteriori to the following criteria: C_0–60_, H_0–210_, O_0–60_, N_0–4_, S_0–2_, P_0–4_ excluding ^13^C isotopologues,^40^ H/C ratio = 0.3 to 3, O/C ratio = 0 to 1. For each time point, formulas detected in instrument and process blanks (SPE extraction of Milli-Q water) were removed, as well as any duplicate assigned MF within a given treatment. The double bond equivalent (DBE) and modified aromaticity index (AI_mod_) were calculated according to Koch and Dittmar,^20,41^ where C, H, O, N, S, and P indicate the numbers of each corresponding atom for a given molecular formula:

1 DBE=1+0.5(2C−H+N+P)

$$AI_{\mathrm{mod}}=\frac{1+C-0.5O-S-0.5\left(N+P+H\right)}{C-0.5O-S-N-P}$$ 2

DBE should be an integer, representing the absence of radical ions. Negative AI_mod_ values were considered zero. DBE, AI_mod_, molecular weight (MW), and H/C and O/C ratios were calculated for all MFs per sample and used to classify molecules into different chemical class groups varying in their level of recalcitrance (Table 1). Carboxyl-rich alicyclic molecules (CRAM) represent a class of potentially recalcitrant components, resistant to biodegradation, and were also identified.^42^ The molecular lability boundary (MLB)^43^ was calculated for all MFs with H/C ≥ 1.5 (MLB_L_) and H/C < 1.5 (MLB_R_). To provide a representative characterization of bulk DOM composition and compare between different samples, peak intensities were normalized to a total sum of 1 × 10^6^ per sample, after which intensity-weighted averages (wa) were calculated for all molecular compounds and DOM metrics according to eq 3. *I*_*i*_ is the signal normalized intensities for a given formula, *A*_i_ is the DOM metric value for that formula, and *F* is the total number of formulas per sample.

3 $$wa=\frac{\overset{F}{\underset{i=1}{\sum }}I_{i}\cdot A_{i}}{\overset{F}{\underset{i=1}{\sum }}I_{i}}$$

**Table 1:**
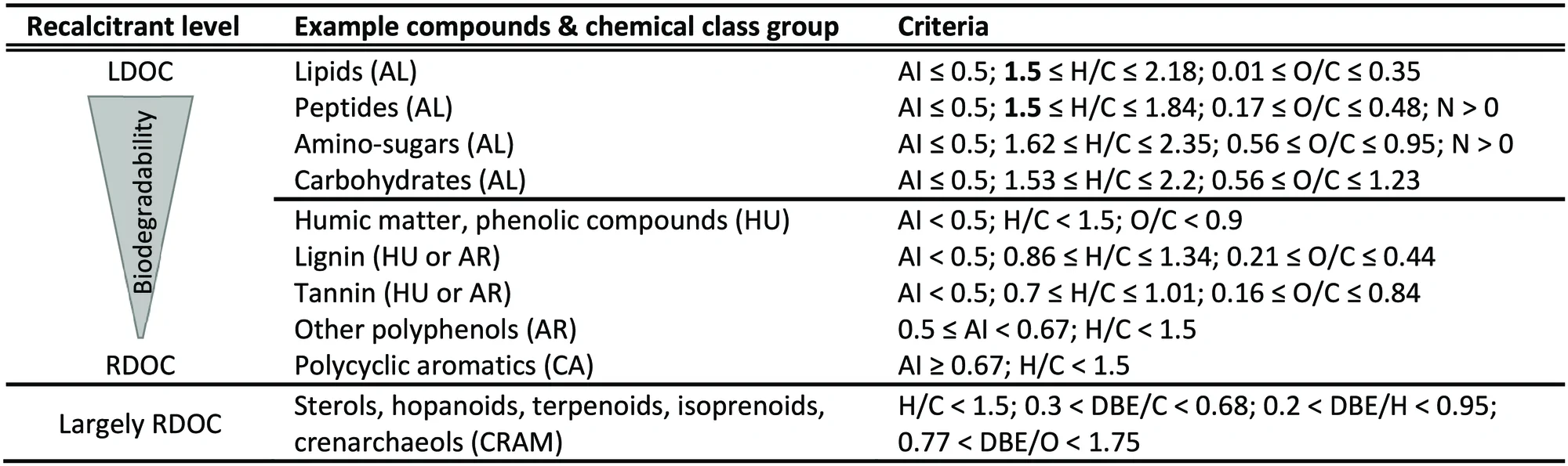
Classification of DOM Compounds into Chemical Class Groups of Varying Recalcitrance Levels^a^ ^,^ ^b^

a *NB*: some chemical class groups may overlap for particular compounds, indicated in parentheses.

b Aliphatic (AL), with a breakdown into 4 example compound groups,^19^ highly unsaturated (HU), aromatic (AR), condensed aromatic (CA),^20–22,41^ and CRAM.^42^ Bold numbers have been modified from the original value^19^ to fit with the MLB concept.^43^

Similarly, intensity-weighted CHONSP, CRAM, MLB_L_, and MLB_R_ (% CHOSNP_wa_, % CRAM_wa_, % MLB_wR_, and % MLB_wR_, respectively) were calculated by adding the normalized peak intensities of all MFs pertaining to each group, divided by the sum of all normalized peak intensities per sample and given as a percentage.

### 2.4 Bacterial Community Composition Analysis

Obtained sequences were treated using Quantitative Insights into Microbial Ecology 2 (QIIME2) pipeline.^44^ Initially, forward and reverse reads of each sample were loaded in an appropriate QIIME2 artifact. Quality control (based on median Phred value > 20), denoising, and amplicon sequence variants (ASVs) were conducted using the DADA2 algorithm.^45^ DADA2 was used to merge complementary ASV forward and reverse reads, and ASVs and counts were generated for each sample. Additionally, chimera sequences were screened and filtered based on the DADA2 de novo approach. Next, representative sequences were aligned using MAFFT^46^ and masked, and a phylogenetic tree was generated using FastTree.^47^ Taxonomic assignment to each representative sequence was conducted using classifier, based on the Silva database (v.138)^48^ and specifically pretrained for the primer set region used to amplify sequenced amplicons. ASVs annotated to chloroplast and mitochondria were removed from the data set. Subsequently, the data set was rarefied to 6638 randomly selected sequences for each sample to obtain an even sampling depth for downstream statistical analysis. The chosen threshold was sufficient to retain all 48 samples for further analysis.

### 2.5 Data Processing and Statistical Analysis

Statistical analysis of DOC and POC concentrations and accompanying seawater parameters were performed with ANOVA and Tukey test, and correlations were investigated using Pearsons r in GraphPad Prism 10 with a significance level of *p* < 0.05. Welch’s correction was used when homogeneity of variance was violated. The high-resolution mass spectrometry data was processed using an automated pipeline developed in Python (version 3.10) utilizing the pandas library. Significance tests were performed using ANOVA, while one sample T and Wilcoxon test were used on DOM metrics. Principal component analysis (PCA) was performed on standardized DOM composition, metrics, and abiotic data to identify clustering between samples. Microbial community data treatment and statistical analysis were performed using various R packages including phyloseq,^49^ rstatix,^50^ and vegan.^51^ *Chao1* and *Shannon* diversity indices were used to evaluate sample diversity, and statistical significance was assessed by nonparametric Kruskal–Wallis and pairwise Dunn’s tests. A significance level of *p* < 0.05 was used, accounting for Bonferroni correction. Multivariate principal coordinate analysis (PCoA) was performed to visualize similarities and dissimilarities between samples using the *Bray–Curtis* dissimilarity index, and statistical significance was tested using PERMANOVA.

## 3 Results

### 3.1 Production of DOC and POC

All incubation treatments had higher DOC concentrations compared to the inlet seawater from 80 m in Trondheim fjord (Figure 2a). DOC concentration increased from 201.5 ± 44.6 μM to 261.7 ± 73.5 μM in the kelp_L_ treatment between 5 and 24 h, kept stable to 48 h, and then decreased after 72 h. High variations resulted in no statistically significant differences over time (*p* > 0.05); however, a general trend maintains, with kelp_L_ showing the highest DOC concentrations, significantly different to control_L_ (*p* < 0.05); stable DOC concentrations in kelp_D_ and lowest concentrations in controls (Figure 2a). Both kelp treatments had higher POC concentrations than their controls and ambient seawater after just 5 h, rising steeply in kelp_L_ from 24 to 48 h (56.4 ± 11.7 μM) and to 72 h (67.0 ± 16.9 μM), significantly different from control_L_ (p = 0.0016). There was a small POC increase from 13.9 ± 2.0 μM to 23.8 ± 4.8 μM between 48 and 72 h in control_L_ but virtually no POC changes in the dark treatments (Figure 2b). Dissolved oxygen, pH, and DIC each showed significant differences between kelp_L_ and all the other treatments (*p* < 0.0001, Figure 2c-e). TA was steady throughout the experiment at 2286–2294 μmol/kg, and total dissolved nitrogen (TDN) was significantly lower in the controls than the kelp treatments (*p* < 0.001, Figure 2f). Pearsons r revealed significant correlations between pH, DIC, DO, and POC at the *p* < 0.0001 level; DOC and TDN at *p* < 0.001; and DOC, POC, DIC, pH, and DO at *p* < 0.05 (Table S2).

**Figure 2:**
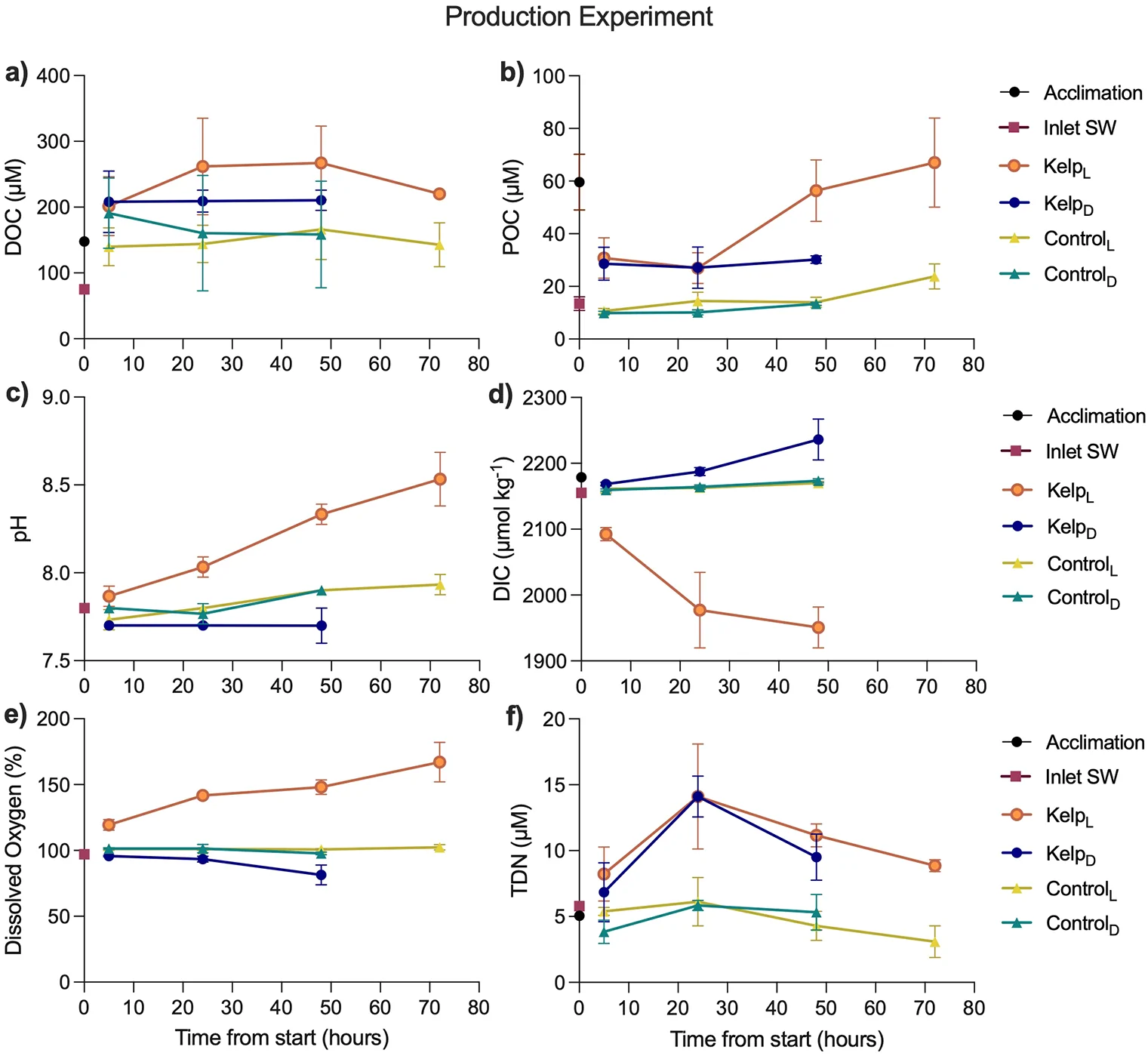
Production of DOC (a) and POC (b) from *S. latissima*, accompanied by abiotic parameters of pH (c), DIC (d), dissolved oxygen (e), and TDN (f), sampled at T_0_ for the inlet SW and acclimation tank, and after 5, 24, 48, and 72 h.

Strong photosynthetic activity and net production were evident in the kelp_L_ incubators (Figure 3a-b), supported by decreasing DIC and increasing DO (*p* < 0.0001, R^2^ = 0.823, Figure 3c). Net respiration was predominant in kelp_D_ accompanied by decreasing DO and increasing DIC (*p* < 0.0001, R^2^ = 0.976) and to a lesser extent in the control treatments (Figure 3b-c). Once corrected for phytoplankton activity, DOC hourly production rates in the light and dark kelp treatments were on average 0.09–1.41 mg C g­(dw)^−1^ h^–1^ versus 0.09–0.25 mg C g­(dw)^−1^ h^–1^, respectively; while POC production rates were on average 0.05–0.46 mg C g­(dw)^−1^ h^–1^ versus 0.03–0.27 mg C g­(dw)^−1^ h^–1^. In light treatments, 4.3 to 6.5% (mean 5.5%) of the of the fixed carbon was released as DOC within 5 and 48 h of production, compared 0.9 to 3.7% (mean 2.7%) in dark treatments. A further 0.7 to 2.4% (mean 1.5%) was lost as small POC fragments (retained in 0.2 μm GF/F filter) compared to 0.9 to 1.3% (mean 1.1%) in light and dark treatments, respectively.

**Figure 3:**
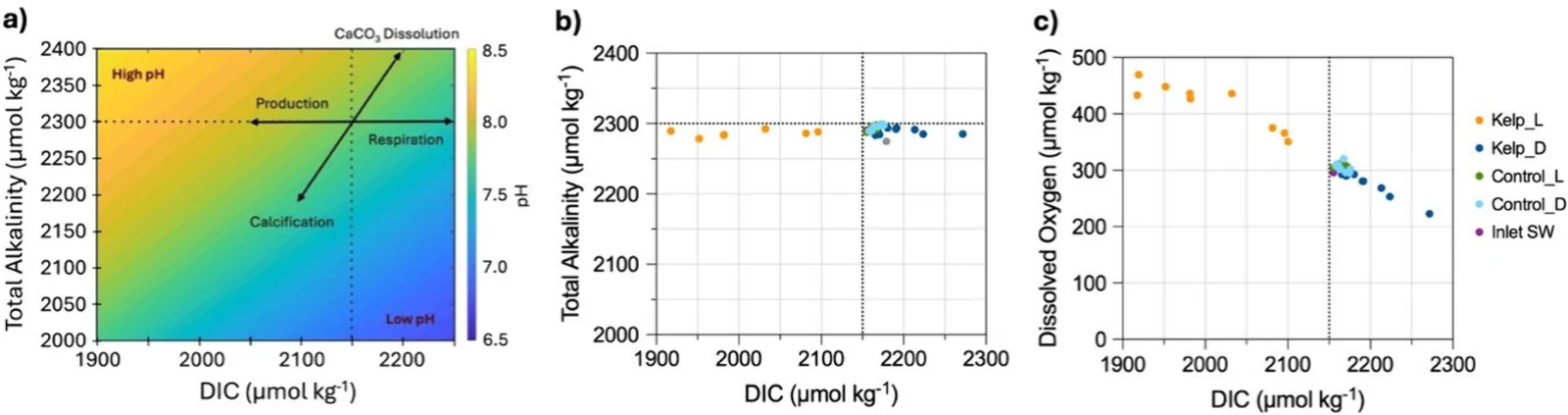
Property-property plots of total alkalinity and dissolved oxygen as a function of DIC, illustrating biochemical processes conceptually (a) and those occurring during the production experiment in different treatments (b, c). Panel (a) reproduced with permission, courtesy of Melissa Melendez (University of Hawaii, USA) and Prof. Andreas Anderson (Scripps Institution of Oceanography, USA).

### 3.2 Biodegradation of Kelp-Derived DOC

DOC biodegradation at 4 and 10 °C is shown in Figure 4a-b, with significant differences between the kelp and control treatments, as well as over time (*p* < 0.0001). Average dissolved oxygen fell from 103.3 to 105.6% to 91.3–92.3% between days 94 and 187 of the biodegradation experiment but was not a limiting factor (Figure S3a-b). The pH increased from 8.33 ± 0.15 to 8.60 ± 0.17 at day 15 (at 10 °C) and 8.40 ± 0.17 to 8.60 ± 0.17 at day 60 (at 4 °C), declining steadily thereafter to 8.08 ± 0.14 and 8.18 ± 0.19, respectively (Figure S3c-d). The pH was significantly higher in kelp_L_ than control_L_ treatments for both temperature conditions (p = 0.0022). Pearsons r revealed significant correlations between DOC and pH at the *p* < 0.0001 level and DO versus pH, DOC, and TDN at *p* < 0.05 (Table S3).

**Figure 4:**
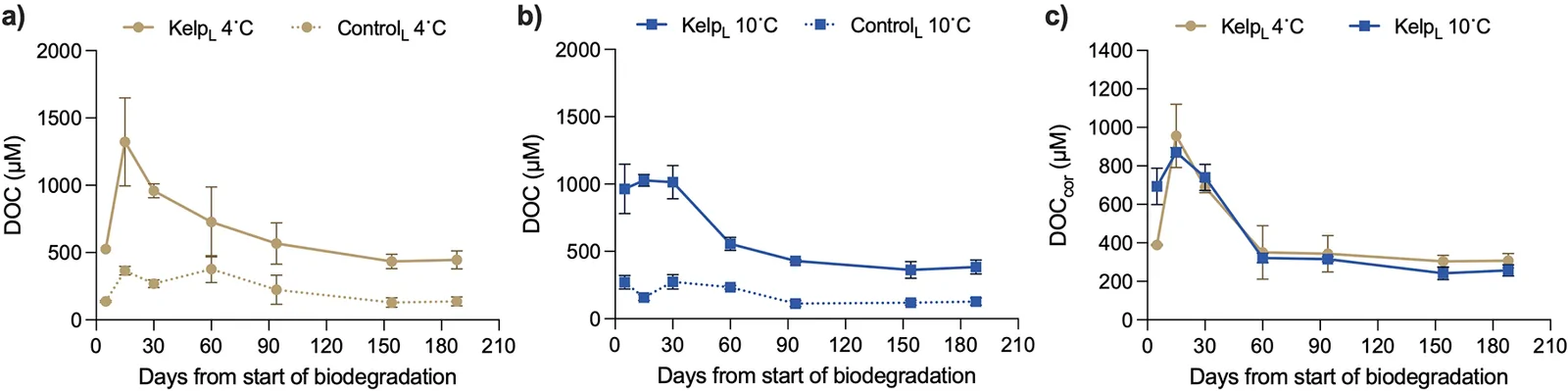
Biodegradation of DOC from *S. latissima* at (a) 4 °C, (b) 10 °C, and (c) corrected for phytoplankton activity (kelp-control).

DOC concentrations were corrected to account for phytoplankton activity and verify the DOC contribution of the macroalgae alone, by subtracting control from kelp values (DOC_cor_). No significant differences were then observed between the two temperatures (p = 0.9192). Average DOC_cor_ concentrations peaked at 871–957 μM after 15 days, and the majority of the DOC was consumed by day 60, reaching 321–351 μM (Figure 4c). Between 60 and 154 days, concentrations slowly dropped by 47–79 μM, whereby stabilization began to occur. The DOC remaining after 187 days was 257–307 μM or 37–79% (mean 58%) from initial DOC levels (Figure 4c).

### 3.3 DOC Molecular Composition and Recalcitrance

The data processing described resulted in a total of 5,303 unique MFs, constituting 70% of the original data set (blanks removed). 1,951 MFs (37%) were unique to the production experiment while 1,805 MFs (34%) were unique to the biodegradation experiment, produced by microbial transformation. 1,547 (29%) MFs occurred in both experiments. CHON, CHOS, and CHONS were 14% higher in the kelp treatments than the controls during the production experiment. The proportion of CHO and CHOP­(N/S) compounds increases gradually throughout the experiments except for kelp_L_ 10 °C after 30 days, which contained higher CHO along with very low amounts of CHOS molecules compared to kelp_L_ 4 °C (Table 2). Compounds in “Other” include nonoxygen containing molecules (e.g., CHS, CHNS) present in marine DOM, products of microbial decomposition, which increased slightly throughout the experiments, though kelp_L_ 10 °C peaked after 30 days. DOM composition in both experimental parts is illustrated in van Krevelen plots (Figure S4.1).

**Table 2:** DOM Molecular Species, Given as Intensity-Weighted Percentages, during Production and Biodegradation

Sampling time	Treatment	% CHO_wa_	% CHON_wa_	% CHOS_wa_	% CHONS_wa_	% CHOP(N/S)_wa_	% OTHER_wa_
**T0**	Inlet SW	50.5	6.1	15.2	6.7	21.0	0.5
**48H**	Kelp_L_	43.5	7.1	21.4	10.0	17.6	0.3
	Kelp_D_	47.5	5.5	18.7	7.6	20.3	0.4
	Control_L_	56.4	6.8	12.9	4.9	18.6	0.4
	Control_D_	58.0	6.2	11.7	5.4	18.2	0.4
**D030**	Kelp_L_ 10 °C	66.5	6.6	4.8	4.1	16.9	1.1
	Kelp_L_ 4 °C	57.6	5.5	12.2	4.4	20.0	0.3
	Control_L_ 10 °C	56.6	6.2	11.2	5.4	20.0	0.6
	Control_L_ 4 °C	58.5	5.9	10.2	5.5	19.1	0.7
**D187**	Kelp_L_ 10 °C	55.0	5.5	12.4	4.9	21.7	0.5
	Kelp_L_ 4 °C	57.2	5.6	10.9	4.9	20.8	0.7
	Control_L_ 10 °C	61.9	5.7	8.4	5.1	18.2	0.6
	Control_L_ 4 °C	56.6	6.1	9.6	6.0	20.9	0.8

The proportions of major chemical groups according to criteria in Table 1 are shown in Figure 5, and these are further compared to CHONSP composition in Table S4. HU compounds were predominant in all treatments, with 73–86% in intensity-weighted relative abundance (Figure 5). They were on average, 3–10% higher in control treatments, and 3% higher in dark than light treatments. AR and CA comprised only 1.5–6% relative abundance throughout both experiments and were, on average, 4% higher in kelp than control treatments during the production experiment. AL had an intensity-weighted relative abundance of 10–22%, highest in kelp_L_ during production, and decreasing twice as much than in control treatments by the end of biodegradation. Lipid-like compounds and other aliphatics (uncategorized) were the most predominant compounds in MLB_L_ (Figure 5). The 659 uncategorized aliphatic compounds represent 139, 33, 16, 48, 419, and 4 MFs in CHO, CHOS, CHON, CHONS, CHOP­(N/S), and “other”, respectively (Table S4).

**Figure 5:**
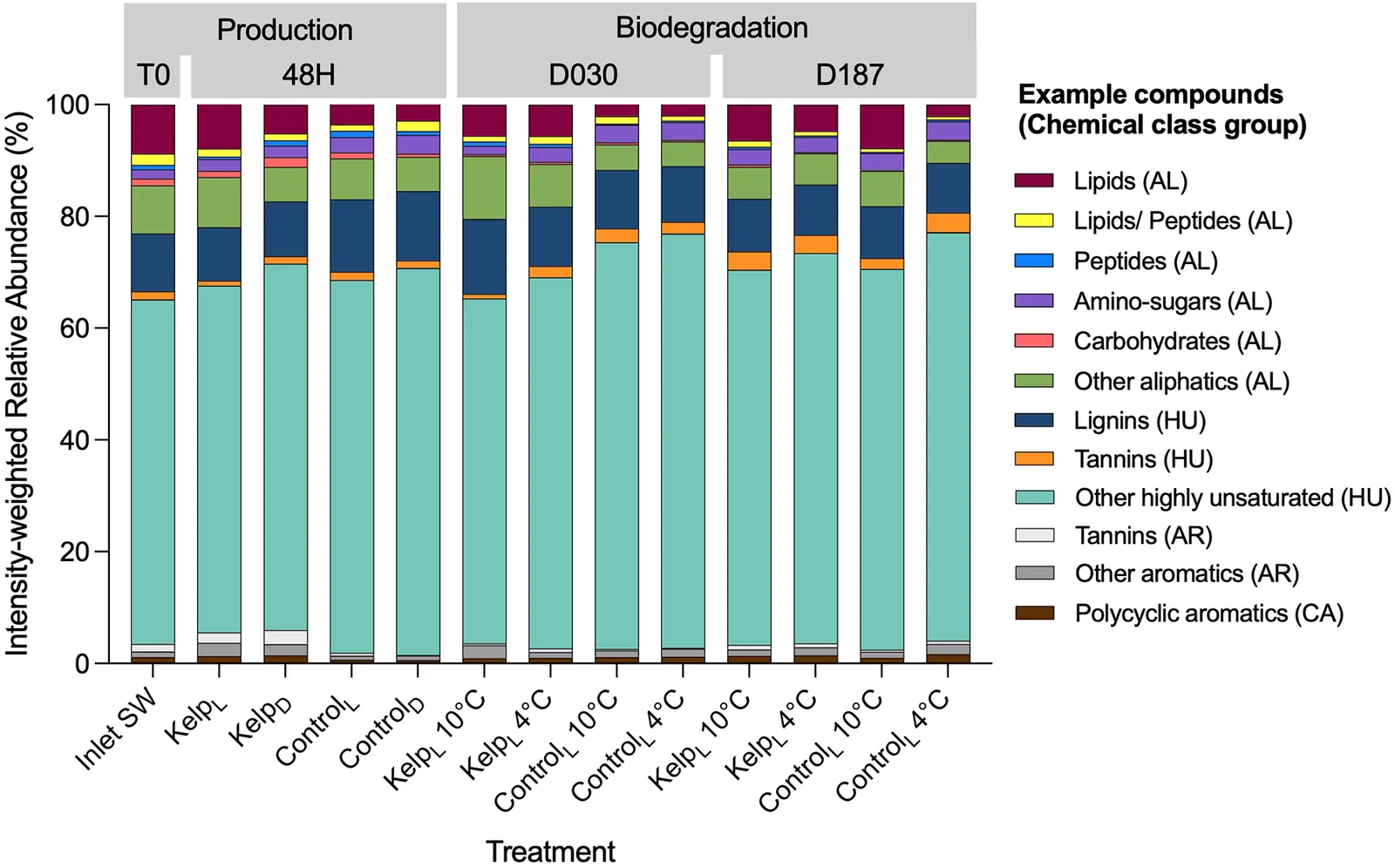
Changes in DOM composition, showing proportions of major chemical class groups and example compounds categorized according to criteria in Table 1: aliphatics (AL), highly unsaturated compounds (HU), aromatics (AR), and condensed aromatics (CA).

During the production experiment, kelp_L_ had the highest number of aliphatic compounds, leading to an average increase of 138 MLB_R_ compounds between the end of the production experiment and D187, compared to 41 in the controls. Intensity-weighted relative abundances of labile compounds (MLB_wL_) were higher in kelp (mean 20%) than control (mean 16%) treatments and higher in light (mean 19%) than dark treatments (mean 16%) (Figure 5, Table 3). Relative abundances in MLB_wR_ increased by 5.1–7.6% in kelp treatments and by 6.6% in control (4 °C); and decreased by 1.2% in control (10 °C). Averaged between temperatures,% MLB_wR_ increased twice as much in kelp than in control treatments by D187.

**Table 3:** DOM Composition, Richness, and Diversity, Including MLB Indices, CRAM Molecules, Total Molecular Formulas (MFs), and Unique MFs Found Exclusively in the Named Treatment

				Richness		Diversity		CRAM	
Sampling time	**Treatment**	**Total MFs**	**Unique MFs**	**% MLB**_**L**_	**% MLB**_**R**_	**% MLB**_**wL**_	**% MLB**_**wR**_	**Av.**nr.	**% CRAM**_**wa**_
**T0**	Inlet SW	1,814	401	27.1	72.9	23.1	76.9	294	42.1
**48H**	Kelp_L_	1,640	441	27.7	72.3	22.0	78.0	281	46.1
	Kelp_D_	1,419	136	23.7	76.3	17.3	82.7	285	47.5
	Control_L_	1,400	51	20.3	79.7	17.0	83.0	321	45.9
	Control_D_	1,395	49	17.0	83.0	15.5	84.5	311	46.7
**D030**	Kelp_L_ 10 °C	1,787	679	23.4	76.6	20.4	79.6	323	48.5
	Kelp_L_ 4 °C	1,467	68	23.3	76.7	18.3	81.7	283	41.6
	Control_L_ 10 °C	1,253	18	14.0	86.0	11.7	88.3	316	45.6
	Control_L_ 4 °C	1,175	13	13.3	86.7	11.0	89.0	303	46.7
**D187**	Kelp_L_ 10 °C	1,438	79	21.0	79.0	16.9	83.1	291	40.6
	Kelp_L_ 4 °C	1,345	25	18.1	81.9	14.3	85.7	297	43.0
	Control_L_ 10 °C	1,609	112	21.4	78.6	18.2	81.8	319	44.0
	Control_L_ 4 °C	1,163	44	12.5	87.5	10.4	89.6	301	45.6

Of the CRAM compounds identified, 99.2% overlapped with MLB_R_, and only 0.8% overlapped with MLB_L_. CRAM molecules comprised 34.0–40.7% of total MFs during both experimental parts, yet intensity-weighed CRAM proportions were 40.6–48.5%; 1–2% higher in dark than light treatments during production and 2.6–5% higher in controls compared to kelp treatments during biodegradation. Despite a small decrease in % CRAM_wa_ throughout the experiments, the average number of CRAM molecules increases slightly in kelp treatments compared to a small decrease in control treatments (Table 3). The number of CRAM and MLB_R_ molecules is shown relative to changes in DOC concentrations during the biodegradation experiment in Figure 6.

**Figure 6:**
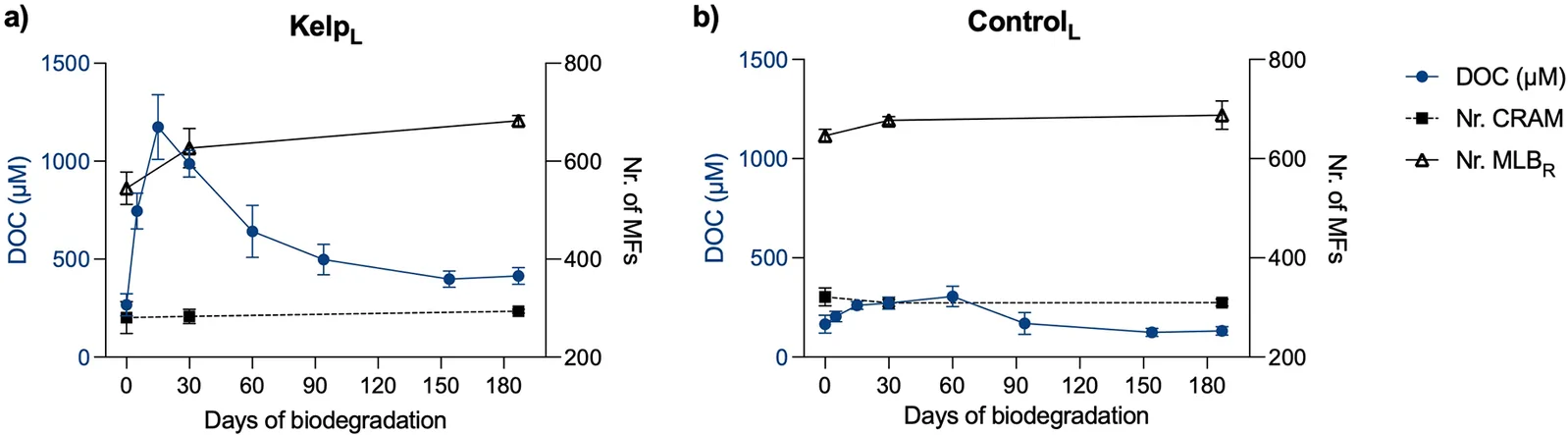
Evolution of DOC concentrations, number of CRAM molecules, and number of molecular formulas in MLB_R_ during biodegradation in kelp and control treatments (averaged for both temperatures). The spike in number of CRAM at kelp_L_ 10 °C (D030) was not included.

To further discern the contribution of the macroalgae from background seawater used to fill the incubators (Figure 7a) and other sources of DOM, unique MFs for each treatment and sample were inspected. MFs present in kelp treatments during the production experiment, not present in the inlet SW or controls, were considered to be uniquely produced by *S. latissima* (Figure 7b). MFs present in the controls but not in the inlet SW were considered contributions from other photoautotrophs (such as phytoplankton, Figure 7c); and those present during biodegradation but not in the production experiment or inlet SW originate from microbial transformations of the DOM (Figure 7d). Using normalized peak intensities previously calculated (cf. 2.3), unique contributions by the 4 named groups accounted for 28.5% of the DOM (Figure 7).

**Figure 7:**
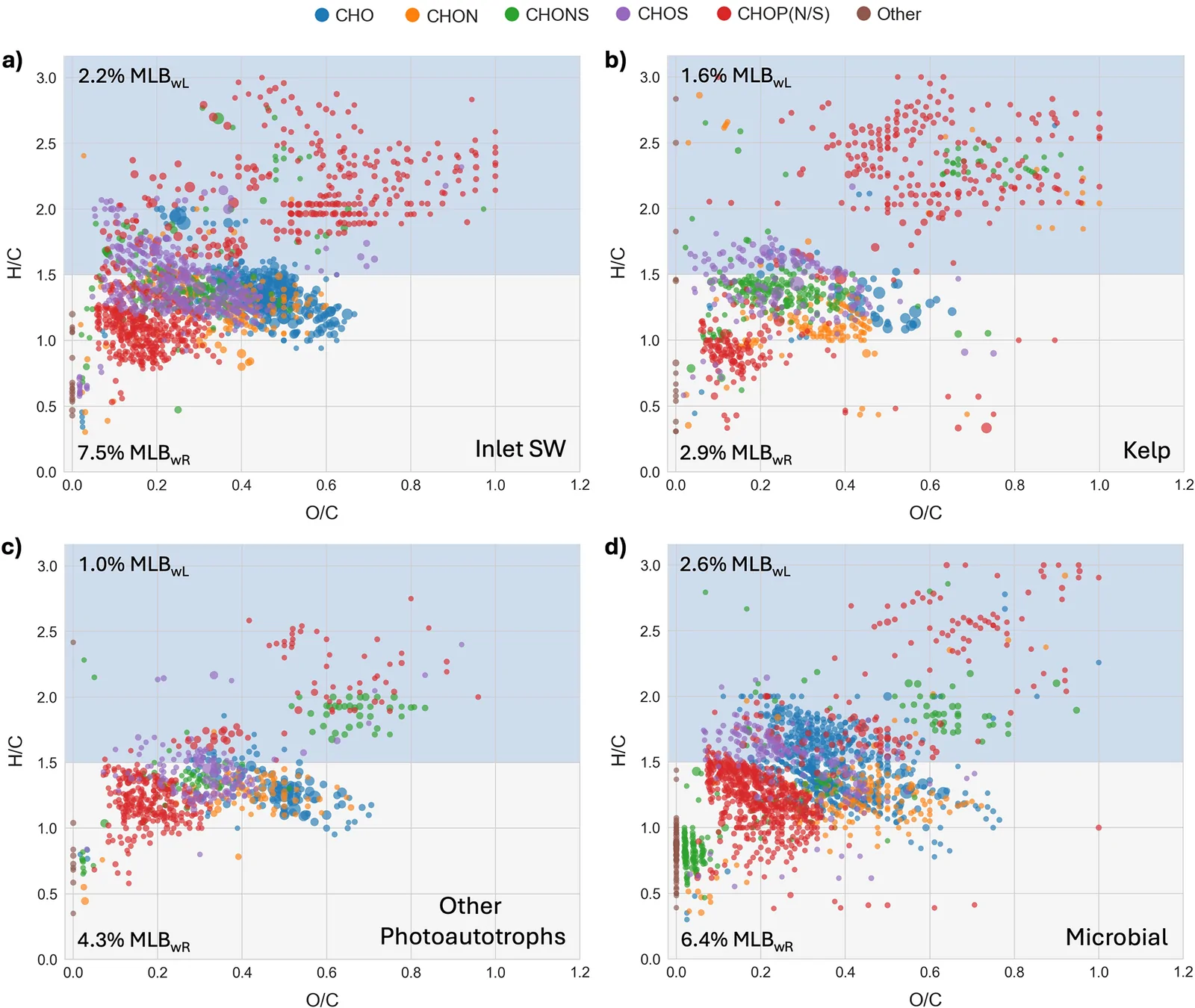
Intensity-weighted abundances for unique DOM molecular formulas in inlet seawater (a) produced by *S. latissima* (b), other photoautotrophs (c), and microbial transformations (d). Intensity-weighted MLB indices indicate proportions of more labile and more recalcitrant fractions from each group, totalling 28.5%.

Intensity-weighted averages for structural DOM metrics calculated across different time points and treatments for both experimental parts are summarized in Table 4 and Figure S4.2. During the production experiment, MW_wa_, DBE_wa_, AI_mod wa_, and the O/C _wa_ ratio were higher in kelp_D_ than kelp_L_, while the H/C _wa_ ratio was lower. Throughout both experiments there is a significant increase in DBE_wa_, AI_mod wa_, and the O/C_wa_ ratio both for kelp and control treatments, accompanied by a significant decrease in the H/C_wa_ ratio (*p* < 0.001). MW_wa_ shows a decreasing trend in the control groups but no clear trends in the kelp groups. The control 10 °C treatment at 187 days was contradictory in trends for all metrics.

**Table 4:** Molecular Structure Characteristics of DOM, Given as Intensity-Weighted Averages, for Treatments at Start (T0) and End (48H) of the Production Experiment, Early Biodegradation (Day 30), and End of the Biodegradation Experiment (Day 187)

Time	Treatment	MW_wa_	O/C_wa_	H/C_wa_	DBE_wa_	AI_mod wa_
**T0**	Inlet SW	549.47	0.38	1.39	9.69	0.20
**48H**	Kelp_L_	533.14	0.38	1.39	9.56	0.20
	Kelp_D_	545.02	0.40	1.35	10.14	0.21
	Control_L_	550.64	0.39	1.36	10.05	0.20
	Control_D_	553.77	0.39	1.35	10.28	0.20
**D030**	Kelp_L_ 10 °C	524.60	0.39	1.36	9.92	0.21
	Kelp_L_ 4 °C	533.45	0.39	1.35	9.95	0.20
	Control_L_ 10 °C	543.53	0.40	1.30	10.68	0.22
	Control_L_ 4 °C	540.14	0.41	1.30	10.74	0.22
**D187**	Kelp_L_ 10 °C	533.09	0.40	1.32	10.37	0.21
	Kelp_L_ 4 °C	530.96	0.40	1.30	10.57	0.22
	Control_L_ 10 °C	527.78	0.39	1.34	10.11	0.21
	Control_L_ 4 °C	538.55	0.40	1.28	11.02	0.23

PCA was performed to verify differences in bulk DOM composition between treatments during the production and biodegradation experiments (Figure 8). Loadings for PC1 and PC2 are given in Figure S4.3. In the production experiment, samples clustered by treatment type, clearly distinguishing kelp_L_ from kelp_D_ and all the controls (Figure 8a). Clustering was less evident in the biodegradation experiment, but some separation can be seen between kelp and control treatments (except control 10 °C at 187 days). Compounds in “Other” (e.g., CHS, CHNS) were higher in kelp 10 °C after 30 days, largely distinguishing it from kelp 10 °C after 187 days and kelp 4 °C (Figure 8b). Differences between replicates are indicative of bottle effects.

**Figure 8:**
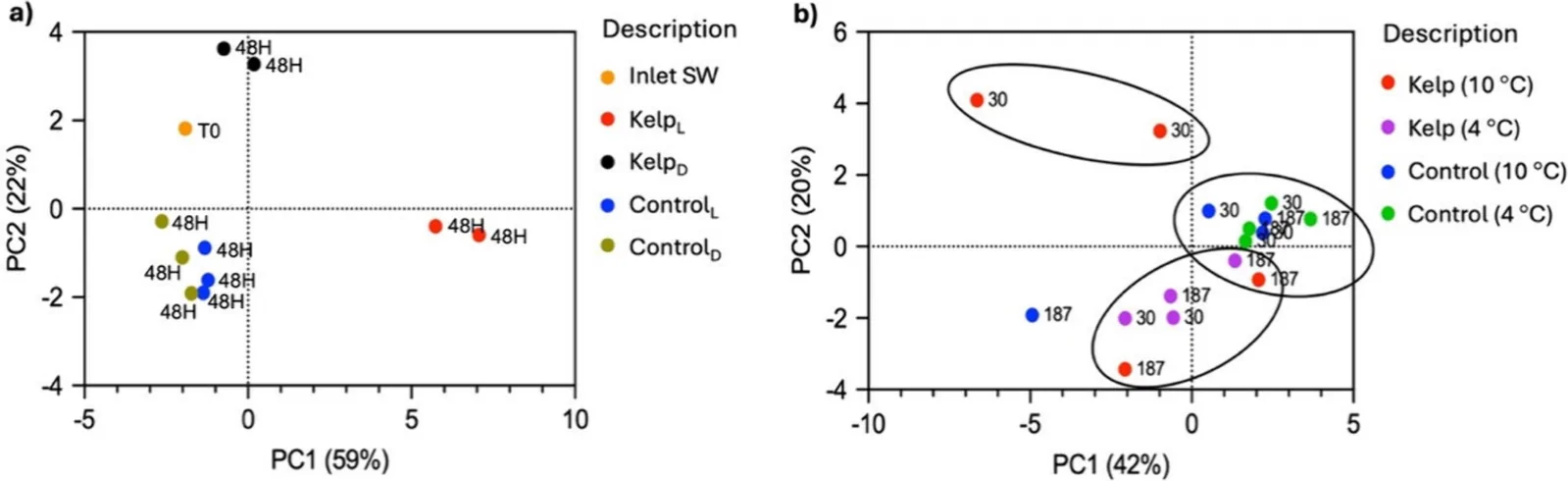
Principal component analysis for DOM composition and abiotic factors during the production (a) and biodegradation (b) experiments. Numbers inside panels indicate sampling hours in (a) and days in (b).

### 3.4 Bacterial Community and Abundance

Bacterial analysis rendered 1451 ASVs, comprising 26 phyla, 46 classes, 126 orders, and 369 genera (Figure S5.1), with dominant classes and orders shown in Table 5. Only 10% of orders were shared between kelp and control treatments at T_0_, 39% at the end of the production experiment (48 h), and 10%, 13%, and 32% after 15, 30, and 187 days of biodegradation, respectively. These trends resulted in significant temporal changes throughout both experimental parts (*p* < 0.05).

**Table 5:** Relative Abundance (%) of Predominant Classes and Orders in Inlet Seawater (Inlet SW), Acclimatization Tank (Acclim.), Kelp Surface Tissue (Kelp S), Kelp Water (Kelp), and Control Water (Control) Treatments

	Production Experiment								Biodegradation Experiment					
	T0		48H - Light			48H - Dark			D015		D030		D187	
TOP CLASSES and ORDERS	Inlet SW	Acclim.	Kelp S	Kelp	Control	Kelp S	Kelp	Control	Kelp	Control	Kelp	Control	Kelp	Control
**Alphaproteobacteria**	**2.0**	**26.2**	**27.5**	**26.9**	**3.6**	**31.8**	**33.1**	**17.7**	**54.1**	**39.2**	**55.5**	**46.9**	**34.8**	**35.3**
Caulobacterales	1.6	4.3	5.6	3.0	1.5	8.3	2.6	10.7	0.3	6.6	0.8	14.4	4.7	8.0
Rhizobiales	0.0	5.6	3.7	4.1	0.1	3.8	3.0	0.3	0.1	0.1	1.0	0.1	2.8	0.9
Rhodobacterales	0.3	12.4	17.3	18.6	1.0	18.9	27.0	5.0	53.3	31.3	53.3	31.3	19.3	8.0
Rhodospirillales	0.0	0.4	0.0	0.1	0.5	0.0	0.1	1.3	0.3	1.2	0.3	1.0	3.5	7.7
Sphingomonadales	0.0	0.6	0.6	0.6	0.0	0.4	0.2	0.1	0.0	0.0	0.1	0.1	0.5	5.4
**Bacteroidia**	**0.8**	**13.2**	**9.9**	**20.2**	**5.2**	**10.0**	**10.1**	**7.0**	**30.0**	**25.2**	**30.8**	**33.9**	**18.3**	**19.6**
Flavobacteriales	0.8	11.5	8.7	20.0	5.2	8.4	10.0	6.9	29.6	25.2	30.4	33.9	16.7	19.2
**Gammaproteobacteria**	**96.9**	**54.4**	**62.1**	**52.4**	**89.6**	**56.8**	**56.3**	**73.4**	**15.9**	**35.5**	**12.9**	**19.2**	**42.7**	**40.4**
Alteromonadales	96.4	42.2	44.5	35.2	79.5	37.9	33.3	64.2	9.8	8.8	2.6	4.4	3.3	1.8
Burkholderiales	0.0	0.2	0.0	0.1	0.2	0.0	0.0	0.3	0.4	25.3	2.2	10.0	11.8	8.7
Cellvibrionales	0.0	1.4	0.4	0.3	0.1	0.4	0.1	0.4	0.1	0.5	2.0	3.2	14.5	9.2
Oceanospirillales	0.3	1.5	0.2	6.3	8.4	0.9	4.5	7.1	4.6	0.7	3.6	0.5	4.9	5.8
Pseudomonadales	0.0	0.9	0.0	5.4	0.0	0.0	4.7	0.2	0.3	0.1	2.3	0.6	7.0	14.0
Vibrionales	0.1	1.1	14.5	4.0	0.2	14.5	13.1	0.1	0.8	0.0	0.2	0.0	0.2	0.1
**Other**	**0.3**	**6.3**	**0.6**	**0.6**	**1.6**	**1.4**	**0.4**	**1.9**	**0.0**	**0.0**	**0.9**	**0.0**	**4.2**	**4.7**

In the production experiment, PCoA and PERMANOVA show significant differences in bacterial community composition among kelp surface tissues, control, and kelp water (*p* < 0.01, Figure 9a). Inlet SW and the acclimation tank were excluded from PERMANOVA analysis due to low number of replicas; however, they share similarities with the control and kelp water groups, respectively (Figure 9a). A diverse assemblage of bacteria was introduced by the kelp, with Verrucomicrobiales, Chitinophagales, Arenicellales, Granulosicoccales, Micavibrionales, and Cytophagales unique to the kelp surface tissue. *Chao1* diversity was significantly higher in the acclimation tank and lowest in inlet SW; both were significantly different from all other groups except for control_L_ (Figure S5.3a). *Shannon* diversity was similar between kelp surface, kelp water, and the acclimation tank, while the control water treatments and the inlet SW had significantly lower indices, indicating fewer dominant bacterial groups (Figure S5.3b).

**Figure 9:**
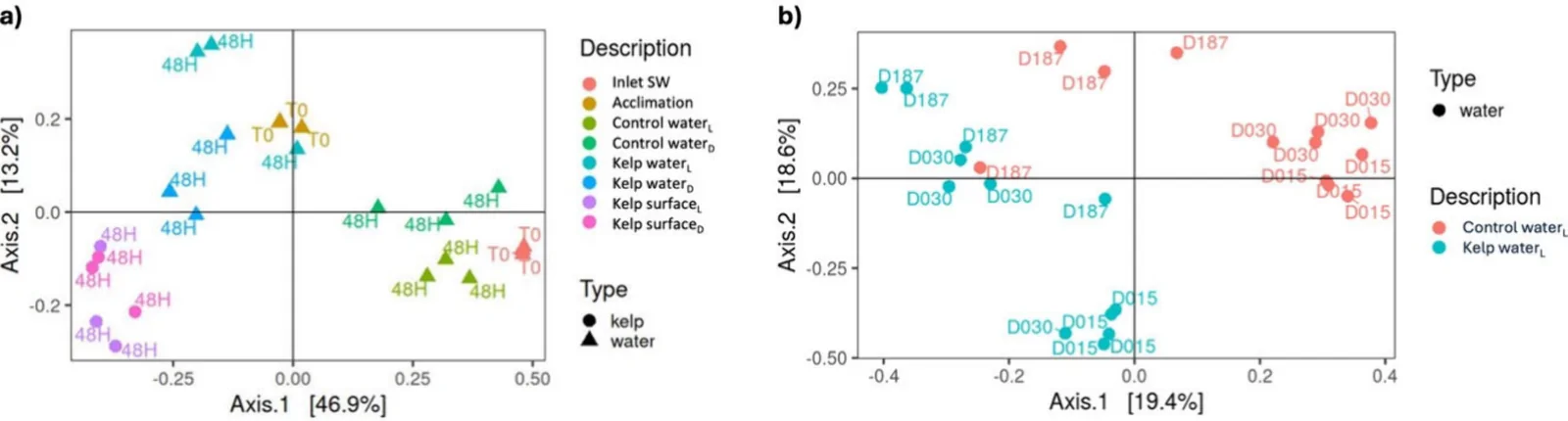
Principal coordinate analysis (PCoA) with Bray–Curtis dissimilarities for bacterial community composition during the production (a) and biodegradation (b) experiments, with numbers inside panels representing sampling times.

During biodegradation, Gammaproteobacteria reduced significantly in relative abundance, while Alphaproteobacteria and Bacteroidia more than doubled. After 187 days, Actinobacteria, Clostridia, Bacilli, and Rhodothermia were characteristic of the sampled water, albeit in small relative quantities. Alteromonadales and Vibrionales (abundant during the production experiment) decreased during the first 30 days of biodegradation, while Rhodobacterales, Flavobacteriales, and Burkholderiales increased (Table 5, Figure S5.2). Significant differences in community composition were observed between kelp water and control water treatments throughout biodegradation (*p* < 0.001, Figure 9b), with significantly higher species richness in kelp water (*Chao1)*; however, *Shannon* diversity, representing species richness and evenness, was not significantly different (Figure S5.3c-d). Community succession led to an increase in average number of orders (14 to 29) and genera (44 to 73) between 15 and 187 days of biodegradation in kelp treatments and slightly less in controls (Figure S5.1). Bacterial groups including Corynebacteriales, Propionibacteriales (Actinobacteria), Kordiimonadales, (Alphaproteobacteria), Sphingobacteriales (Bacteroidia), Lachnospirales, Peptococcales (Clostridia), Balneolales (Rhodothermia) Aeromonadales (Gammaproteobacteria), Bacillales (Bacilli), and Bacteroidales (Bacteroidia) are examples of community succession observed after 187 days (Figure S5.2).

## 4 Discussion

### 4.1 Organic Carbon Release and Biodegradation

Strong photosynthetic activity was evident in kelp_L_, while respiration was predominant in the kelp_D_ (Figures 2, 3). In the first 24 h, average DOC production rates of *S. latissima* were 0.5–1.4 mg C g­(dw)^−1^ h^–1^ in kelp_L_. Published values include 1.41 ± 0.20 mg C g­(dw)^−1^ h^–1^ for *S. latissima* deployed in Winter (January),^52^ 0.12–0.17 mg C g­(dw)^−1^ h^–1^ in an April incubation for *Ecklonia cava*,^12^ and 0.7–1.0 mg C g­(dw)^−1^ d^–1^ reported for species in the genus *Saccharina*.^53^ DIC can be rapidly fixed and released as DOC within just 3–8 h; however, most DOC release results from an excess of fixed carbon relative to the availability of essential nutrients needed for growth, such as nitrogen.^54^ The average fixed carbon released as DOC within 5 and 24 h (4.3 and 6.5% in kelp_L_) is roughly half the reported values from Neves et al.^52^ Possibly, the experimental incubators filled with seawater from an 80 m depth were more nutrient-rich than surface waters at the cultivation location in Hitra, fulfilling the kelp’s metabolic needs with less *excess* carbon to be released as DOC. Similarly, the reduction in average DOC concentrations in the kelp_L_ treatments after 72 h may have resulted from a reduction in excess carbon relative to available nutrients, which were progressively taken up by the kelp and/or possible effects to the kelp’s physiological state. An increase in turbidity and chlorophyll in the experimental basin may also have affected photosynthesis (Figure S1), and/or DOC aggregation into POC may have occurred. DOC and POC pools are intrinsically linked in complex organic transformations mediated by microorganisms, difficult to verify independently.^26,55^

Zhang et al.^32^ describe four stages during DOC biodegradation, also observed in this study, albeit with slightly different time windows: DOC rising (DR); DOC rapidly declining (RD); DOC slowly declining (SD); and DOC stable (DS) (Figure 4). DOC concentrations stabilize at 37–79% (mean 58%) of initial DOC levels after 187 days with no significant differences between temperatures (Figure 4). These levels are in agreement with previous publications from other species, namely, 37.8 to 58% by cultivated *Saccharina japonica*,^17,56^ 56 to 78% from wild *Sargassum horneri*,^15^ but higher than the 24% average reported from species in the genus *Saccharina*.^53^ Differences may be due to differences in experimental protocols, higher temperatures used (22 °C), or bottle effects resulting in different bacterial community assemblages affecting DOC degradation.^53^ Percentages from stable DOC concentrations do not reflect true RDOC, rather the proportion of the DOC which falls in the more recalcitrant fraction (MLB_R_). Furthermore, this fraction has been shown to decrease in 100-year modeled projections (representing semirecalcitrant DOC, SRDOC) by about 13% for the genus *Saccharina* and may be subject to variations due to macroalgae species, differences in DOC composition, microbial community composition, macroalgal physiological state, and environmental conditions.^53^ A similar level of decrease applied to our results would mean ca. 45% of the released DOC could be classified as SRDOC, a meaningful level in climate mitigation efforts.

### 4.2 Molecular Composition and Recalcitrance

81% of the assigned molecular formulas were within the range −10 ≤ DBE-O ≤ 10 representing unequivocal MFs in the DOM data set.^57^ MFs outside this range may contain uncertainties in their assignment; thus analysis of these formulas was performed with caution. FT-ICR analysis revealed that microbial activity began sooner in the dark treatments than those exposed to light in the production experiment (Table 2). In kelp_L_, compounds containing nitrogen, sulfur, or both (CHON, CHOS and CHONS) comprised 38.6% in intensity-weighted relative abundance, preferentially taken up by bacteria for growth.^17,32^ These compounds decreased over 8 times faster in kelp treatments than their controls in the first 30 days of biodegradation. Within CHOS_wa_, which comprised 21.4% of the DOM in kelp_L_ at the end of the production experiment and an average 11.7% in kelp treatments after 187 days of biodegradation, fucoidans are of particular interest as they have been shown to be secreted in high amounts from brown algae and are highly recalcitrant.^58^ Thus, macroalgae may secrete RDOC molecules directly, besides the LDOC which is converted by bacteria into RDOC over time,^17,58^ although it is suggested that microbially produced RDOC has far longer residence times.^27^ Ultimately, DOM reactivity depends on its chemical properties, structure, and environmental conditions affecting vertical mixing and microbial community composition that may remineralize previously accumulated compounds in deeper water layers or allow them to accumulate.^43,59,60^

Unique bacterial groups introduced via the kelp tissue surface led to significant differences in diversity between kelp and control treatments because of community succession. Dominant bacterial classes were in agreement to published literature with macroalgae.^28,32^ In the first 15 days of biodegradation, small amounts of POC still present in the filtered seawater may have been broken down into DOC by polymer hydrolyzers such as Bacteroidia (with 30 and 25% relative abundances in kelp and control treatments) leading to the rise in DOC concentrations observed.^27^ Bacteroidia more than doubled in water samples between the production experiment and 30 days of biodegradation, possibly driving the rapid transformation of compounds (Table 4). Campylobacteria, Alphaproteobacteria, and Bacteroidia are sulfur-degrading bacteria with known metabolic capacity of converting LDOC to SLDOC or SRDOC, and sulfite-reducing bacteria Clostridia and Kapabacteria potentially drive further conversion to RDOC.^27,32^ Campylobacteria and Clostridia were present in very low proportions; however, Alphaproteobacteria constituted around 55% in kelp treatments up to day 30 and 39–47% in the controls at days 15 and 30. A diverse microbial community resulted at the end of biodegradation (Figure S5.2), some of which may have been able to utilize RDOC molecules; however, most were in such low amounts that little such transformations would likely result thereafter.^27^ More likely, LDOC, SLDOC, and any SRDOC molecules still present at day 187 would be converted to RDOC with more time, including more aromatic and condensed aromatic compounds. Bacterial abundance measurements and reinoculation experiments would help verify recalcitrance by assessing any further degradation of the remaining DOM from fresh microbial communities, nutrient replenishment, or exposure to solar radiation.^17^

Similar to *S. japonica*,^17,32^ the majority of the DOM was HU compounds, and AR and CA compounds showed comparatively minor proportions (<10%); however, AL compounds were nearly half in relative abundance.^32^ These compositions represent a mix of DOM sources, which we further discerned into unique contributions from the kelp, other photoautotrophs, inlet SW, and microbial production (Figure 7). 920 out of 5,303 MFs were found exclusively in the DOC released by *S. latissima*, equating to 4.5% intensity-weighted abundance in bulk DOM, with 1.6% and 2.9% in more labile and recalcitrant fractions, respectively. The range in CRAM proportions was slightly lower than reported for *S. japonica* biodegradation^17^ and slightly higher than reported from Trondheim fjord in late spring (May).^40^ A very small decline was observed in % CRAM_wa_ for kelp and control treatments, contrary to Li et al.;^17^ yet the number of CRAM formulas increased in kelp treatments. Meanwhile, labile compounds decreased both in number (MLB_L_) and in relative abundance (% MLB_wL_) (Table 3), and increases in MLB_R_ and % MLB_wR_ were more than double in kelp treatments compared to controls (Figure 6), suggesting a gradual increase in recalcitrance despite small variations in CRAM compounds.

While incubation studies cannot resolve complex environmental interactions affecting DOM recalcitrance, metrics such as DBE, AI_mod_, MW, and H/C and O/C ratios help describe some of its structural and chemical properties. DBE reflects the sum of double bonds and rings in a molecule, while AI_mod_ is a measure of “density” of C–C double bonds, both indicative of greater recalcitrance when higher.^20,41^ Lower molecular weights (LMW) indicate older DOM and lower reactivity/bioavailability,^33,61,62^ as are higher O/C ratios.^63^ H/C ratios are a measure of hydrogen saturation with lower values representative of more recalcitrant molecules, less bioavailable, and more resistant to biodegradation.^43^ Except for control_L_ 10 °C, samples showed an increase in DBE, AI_mod_, and the O/C ratio and a decrease in the H/C ratio. Similar trends were reported in other macroalgae studies after long-term biodegradation, indicating greater recalcitrance of the remaining DOM.^17,27,32^

According to Hansell,^64^ recalcitrant or refractory DOC includes four fractions: SLDOC, SRDOC, RDOC, and URDOC (ultrarecalcitrant), with turnover times for SLDOC within months to years, SRDOC within decades to centuries with a turnover of ca. 20 years, and RDOC and URDOC with lifetimes of ca. 16,000 and 40,000 years, respectively. This means that the MLB_R_ molecular formulas described in this study align with SLDOC in oceanographic research, where compositional changes are still occurring (Figure 5) despite a stabilization in concentrations after 187 days. Within this limited time frame, SRDOC and RDOC could not be quantified and may require modeling approaches similar to that described in Watanabe et al.^53^ There, RDOC was estimated at 2.2% of net primary production (NPP) for species in the genus *Saccharina* over 100-year time scales. Other studies have quantified RDOC as 1.6% from biomass (tissue) carbon from *Ulva prolifera* ^28^ and 0.3%^32^ to 1.27%^27^ from cultivated *S. japonica*, with a further 0.12% of the initial biomass carbon remaining as RPOC.^27^

The concept of island of stability (IOS)^65^ describes a set of organic compounds in marine DOM with the most stable combination of elements in the narrow window of H/C = 1.17 ± 0.13, O/C = 0.52 ± 0.10, and MW between 360 ± 28 and 497 ± 51 Da. Compounds in the IOS persist on geological time scales over 1.5 times the mean residence time of DOC.^65^ 6% of the total number of molecular formulas were identified according to these ranges in the full data set, and 1% remained after 187 days of biodegradation. Kelp treatments showed an intensity-weighted abundance of 0.2% IOS, reflecting the portion of the released DOC with permanence on geological time scales.

DOM characterization often results in overlapping classifications in the van Krevelen space,^19^ and the lack of standardized methods for sampling, analysis, and data processing make comparisons between studies particularly difficult. More empirical criteria would help make more robust distinctions between labile and recalcitrant compounds and perhaps help identify SLDOC or SRDOC compounds. Furthermore, solid phase extraction (SPE) cannot isolate all DOM fractions.^66^ Incubation experiments may constrain bacterial community composition and cause bottle effects, deviating from real-world environmental conditions and interactions affecting DOM reactivity. Despite these limitations, the present study describes some of the processes during production, biodegradation, and recalcitrance of the DOM released from *S. latissima* and suggests IOS analysis may be a suitable indicator of “permanent” RDOC.

### 4.3 Implications for Macroalgae Cultivation

Kelp mariculture areas have been shown to produce significantly higher amounts of DOC and RDOC compared to nonmariculture areas^17,37,56^ and in some cases substantially higher than DOC released by phytoplankton.^56^ The consistent release of DOC and POC from macroalgae during grow-out increases the size and diversity of the DOC pool and gradual production of RDOC by microorganisms. The microbial carbon loop is intrinsically linked to carbon export to deeper ocean layers via the biological pump,^26,28^ contributing to carbon sequestration in the oceans.^2,17,27,67^ It is clear that environmental conditions will lead to region-specific RDOC production potential and that significant scale-up of the industry is needed in Europe together with cost and emission reductions to deliver climate positive solutions.^68–73^ Modeling can be an essential tool to estimate RDOC production within a complex mix of biological, physical, and chemical factors, helping to describe the contribution of macroalgae cultivation to carbon sequestration.^74,75^ Our results provide new data on the production of RDOC for one of Europe’s main cultivars but require replication in real-world contexts. Nonetheless, methods used may help inform methodology, reporting and verification (MRV) frameworks for marine carbon dioxide removal with macroalgae, particularly regarding long-term permanence. These frameworks are increasingly important to address knowledge gaps and create robust carbon accounting^76^ to meet stakeholder needs, including the IPCC, which recently mandated new guidance to incorporate seaweed within national greenhouse gas inventories.^77^

## Supplementary Material



## Notes

Additional experimental details for production and biodegradation experiments; multiparameter statistical correlations; van Krevelen plots; graphical illustration of DOM metrics - MW, DBE, AI_mod_, H/C and O/C ratios; loadings for principal components in PCA analysis; bacterial community composition (full data set illustrations); *Chao1* and *Shannon* diversity indices (PDF)

## #

S.A.: Sensor Globe AS, 0152 Oslo, Norway

## ○

A.B.: Climate and Environment, SINTEF Ocean AS, Sealab, 7010 Trondheim, Norway.
