# Supplementary material for: Production, Biodegradation, and Recalcitrance of Organic Carbon from Cultivated Saccharina latissima in Norway
Source: Environ Sci Technol. 2026 Aug 11;60(33):23268–81. doi: 10.1021/acs.est.6c05450 (PMC13523695; doi:10.1021/acs.est.6c05450)
Supplement: Supplementary file 1 [file es6c05450_si_001.pdf]

Appendix A: Supporting Information

Production, biodegradation and characterization of organic carbon from cultivated *Saccharina latissima* in Norway

Luiza Neves* [a,b], Sebastian Aker[c,#] , David Aldridge[b], Deni Ribičić[d], Anders Brunsvik[e,$] , Ole Jacob Broch[b], Jorunn Skjermo[b] and Murat Van Ardelan[a]

[a] Department of Chemistry and Biomedical Science, Norwegian University of Science and Technology NTNU, NO-7491 Trondheim, Norway

[b] Fisheries and New Biomarine Industries, SINTEF Ocean AS, Sealab, 7010 Trondheim, Norway

[c] Department of Biology, Norwegian University of Science and Technology NTNU, NO-7491Trondheim, Norway

[d] Aquaculture, SINTEF Ocean AS, Sealab, 7010 Trondheim, Norway

[e] Biotechnology and Nanomedicine, SINTEF Industry AS, NO-7465 Trondheim, Norway

*Email: luiza.neves@sintef.no

Summary

Number of figures: 8

Number of tables: 3

Pages: 6

SI.1. Ambient basin conditions during the Production experiment.

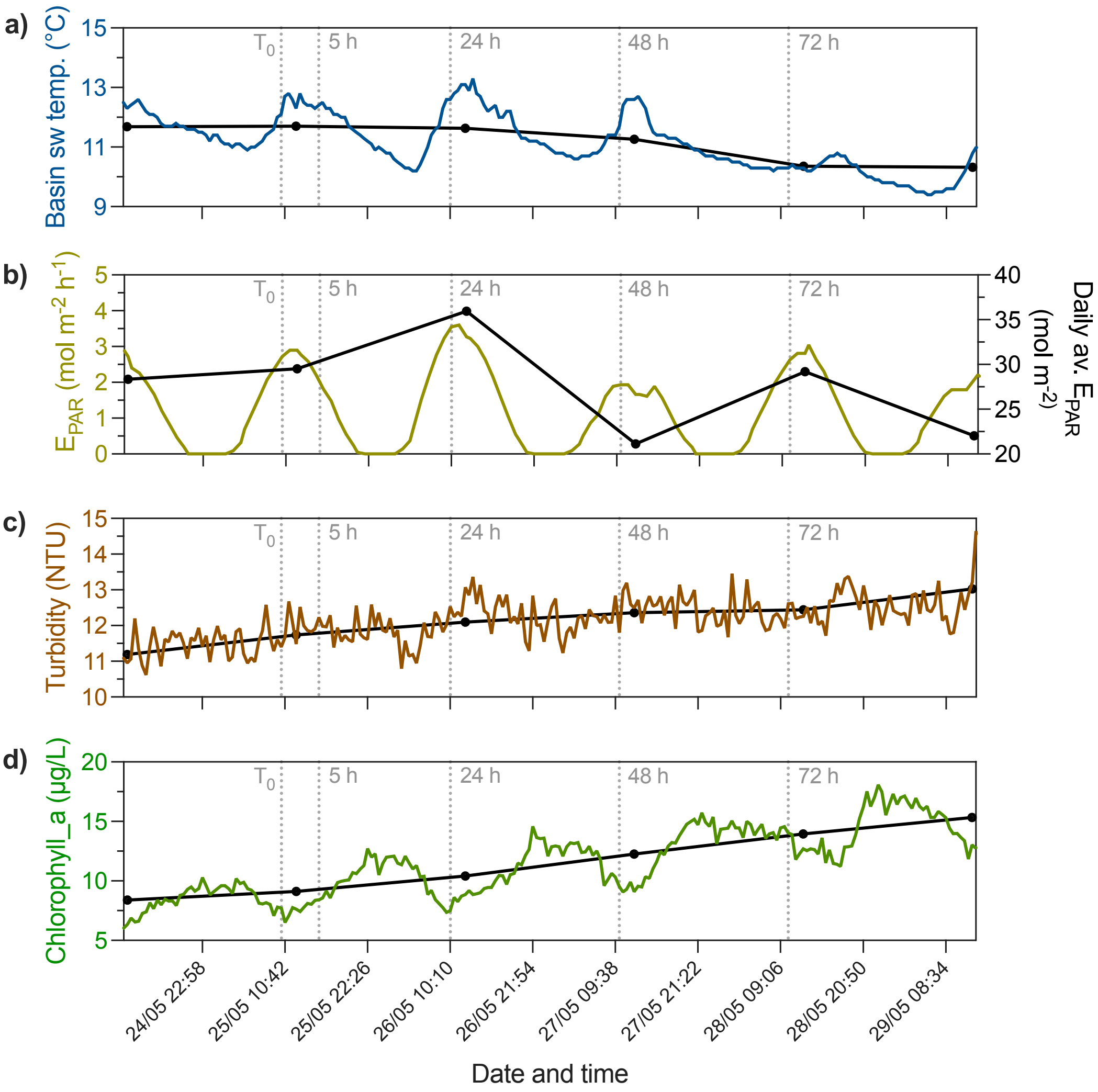

Figure S1: Ambient conditions inside the experiment basin, showing seawater temperature (a), surface light intensity (E_{PAR}) from natural daylight (b), turbidity (c) and chlorophyll (d). Daily averages shown as black lines in each panel, and dotted grey lines represent the sampling times. E_{PAR} data obtained from NASA Power satellite dataset "All Sky Surface Photosynthetically Active Radiation" (available online, accessed on Sept 2025) and a conversion factor of 4.61.

SI.2. Multi-parameter correlations: Production experiment

Table S2: Pearson r correlation analysis between multiple variables during the production experiment, showing p-values (output from Graphpad Prism 10). Green indicates p<0.05, yellow indicates p<0.001 and blue indicates p<0.0001.

**P-VALUES**	Temp. (°C)	pH	Salinity	TA (µmol/kg)	DIC (µmol/kg)	DO (µmol/kg)	POC (µmol/L)	DOC (µmol/L)	TDN (µmol/L)
Temp. (°C)		0.1890	6.1128E-06	0.0178	0.6531	0.7588	0.8579	0.1828	0.4860
pH	0.1890		0.0879	0.1923	1.0835E-11	9.8178E-19	1.0933E-08	0.0278	0.2094
Salinity	6.1128E-06	0.0879		0.0148	0.5706	0.5973	0.7574	0.8685	0.1810
TA (µmol/kg)	0.0178	0.1923	0.0148		0.0537	0.0407	2.3378E-07	0.2053	0.2663
DIC (µmol/kg)	0.6531	1.0835E-11	0.5706	0.0537		1.472E-25	0.0009	0.0082	0.0057
DO (µmol/kg)	0.7588	9.8178E-19	0.5973	0.0407	1.472E-25		5.0756E-08	0.0053	0.0176
POC (µmol/L)	0.8579	1.0933E-08	0.7574	2.3378E-07	0.0009	5.0756E-08		0.0348	0.0104
DOC (µmol/L)	0.1828	0.0278	0.8685	0.2053	0.0082	0.0053	0.0348		0.0008
TDN (µmol/L)	0.4860	0.2094	0.1810	0.2663	0.0057	0.0176	0.0104	0.0008	

SI.3. Biodegradation experiment parameters and correlations

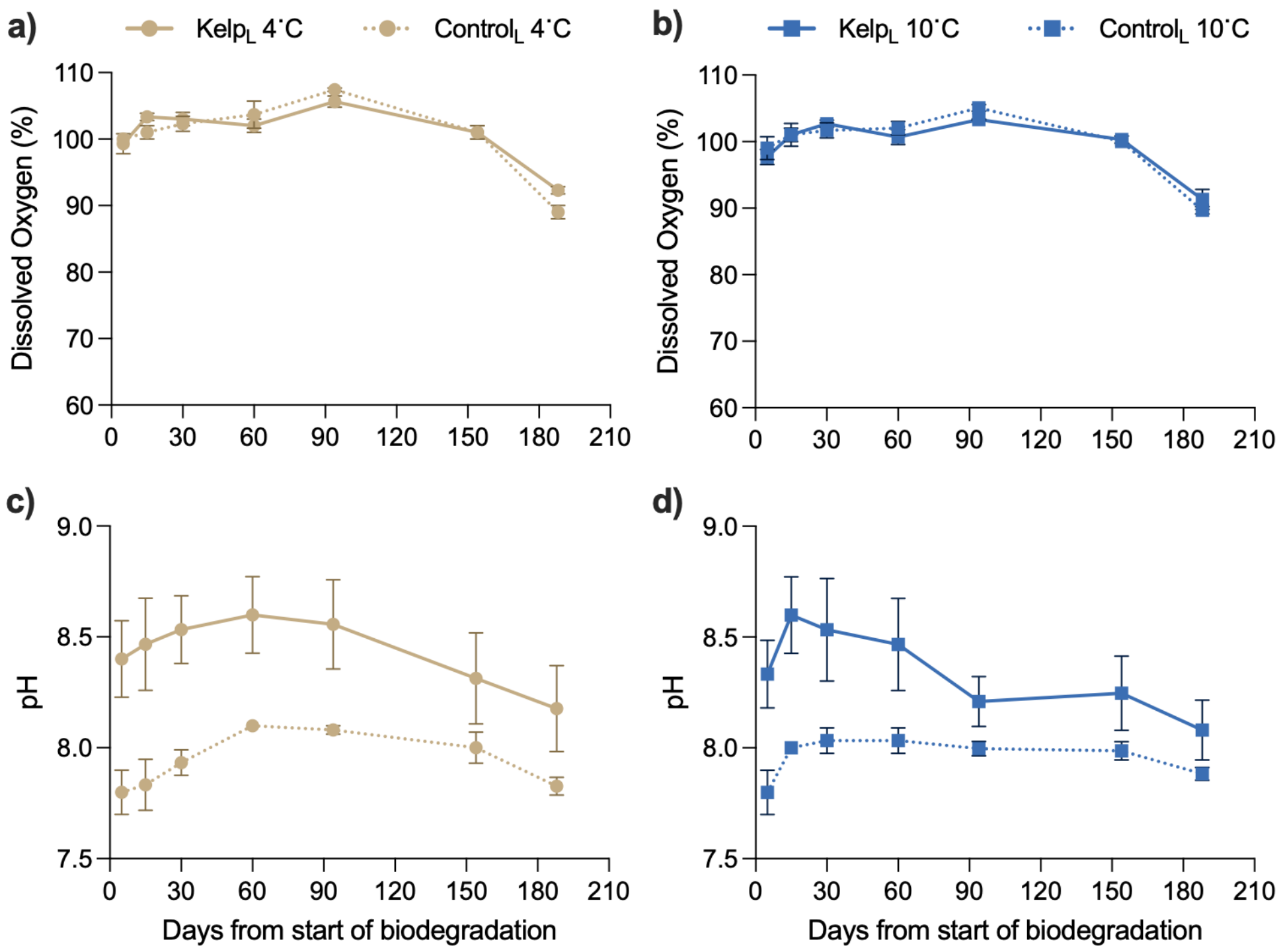

Figure S3: Dissolved oxygen (a, b) and pH (c, d) sampled during 187 days of the biodegradation experiment at 4 and 10 °C.

Table S3: Pearson r correlation analysis between multiple variables during the biodegradation experiment, showing p-values (output from Graphpad Prism 10). Green indicates p<0.05 and blue indicates p<0.0001.

**P-VALUES**	Temperature (°C)	pH	DO (µmol/kg)	POC (µmol/L)	DOC (µmol/L)	TDN (µmol/L)
Temp		0.6285	0.9715	0.1239	0.6758	0.5710
pH	0.6285		0.0047	0.3809	5.8191E-08	0.8451
DO (µmol/kg)	0.9715	0.0047		0.7054	0.0016	0.0118
POC (µmol/L)	0.1239	0.3809	0.7054		0.1677	0.6269
DOC (µmol/L)	0.6758	5.81914E-08	0.0016	0.1677		0.8253
TDN (µmol/L)	0.5710	0.8451	0.0118	0.6269	0.8253	

SI.4. DOM molecular composition

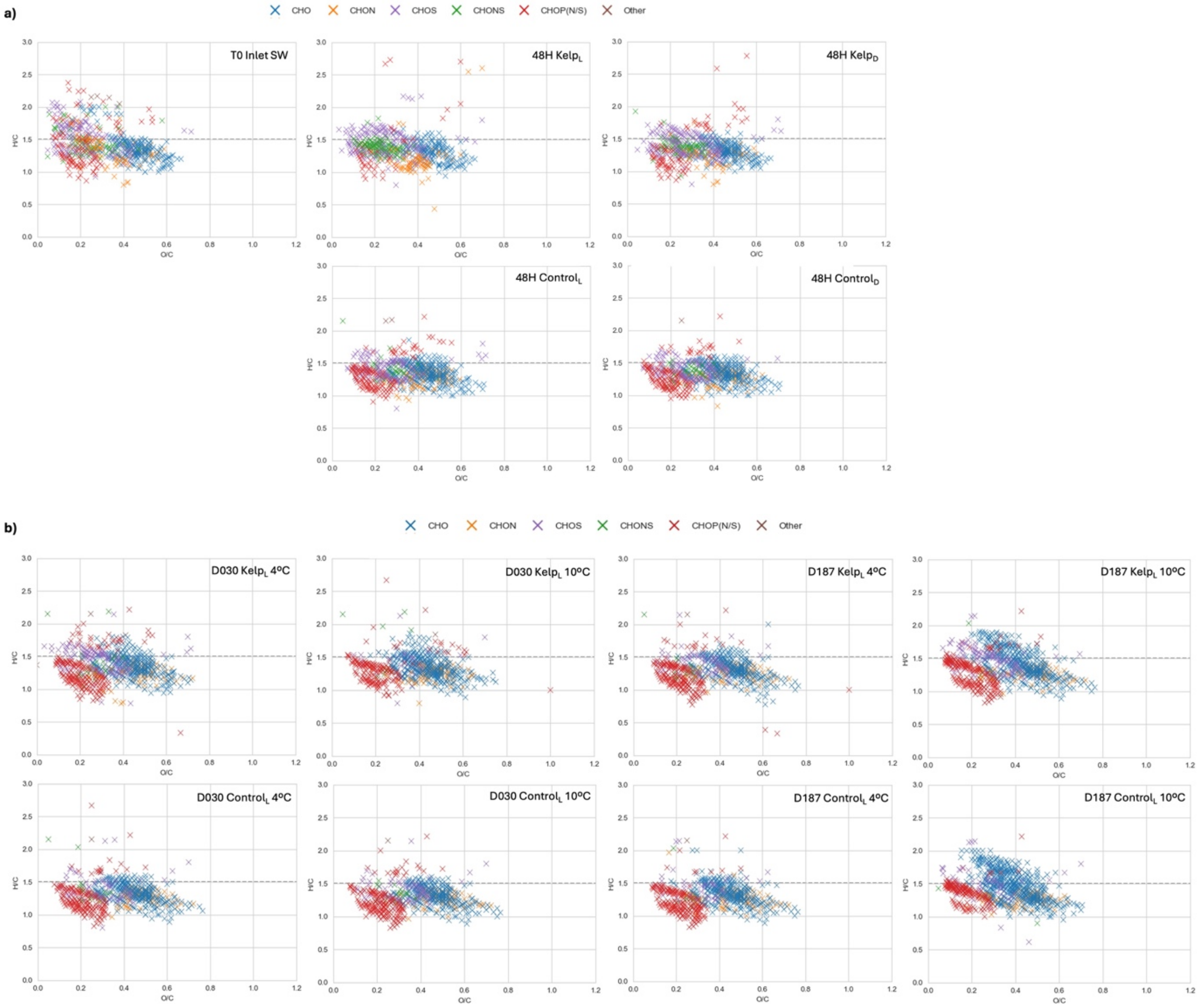

Figure S4.1: van Krevelen plots showing DOC composition changes during the production (a) and biodegradation (b) experiments. The molecular lability boundary, MLB = 1.5 (D'Andrilli et al 2015), is shown in dotted lines, separating more labile from more recalcitrant compounds.

Table S4: DOM classification according to chemical class groups and molecular species for all molecular formulas from both production and biodegradation experiments. Overlaps between certain chemical class groups are illustrated.

Example compounds (Chemical class group)	Molecular species						**Total compounds**
	CHO	CHOS	CHON	CHONS	CHOP(N/S)	Other	
Lipids (AL)	205	319	5	30	89	0	**648**
Lipids/ Peptides (AL)	0	0	17	31	46	0	**94**
Peptides (AL)	0	0	4	4	40	0	**48**
Amino-sugars (AL)	0	0	7	94	98	0	**199**
Carbohydrates (AL)	6	11	4	1	79	0	**101**
Other aliphatics (AL)	139	33	16	48	419	4	**659**
Lignins (HU)	181	187	0	0	9	0	**377**
Tannins (HU)	18	2	28	3	71	0	**122**
Lignins/ Tannins (HU)	3	2	0	0	6	0	**11**
Other highly unsaturated (HU)	494	241	422	396	940	15	**2,508**
Tannins (AR)	1	4	12	2	92	0	**111**
Lignins/ Tannins (AR)	0	0	0	0	7	0	**7**
Other aromatics (AR)	3	12	9	89	124	42	**279**
Condensed aromatics (CA)	10	17	24	19	36	33	**139**
**Total per chemical class**	**1,060**	**828**	**548**	**717**	**2,056**	**94**	**5,303**

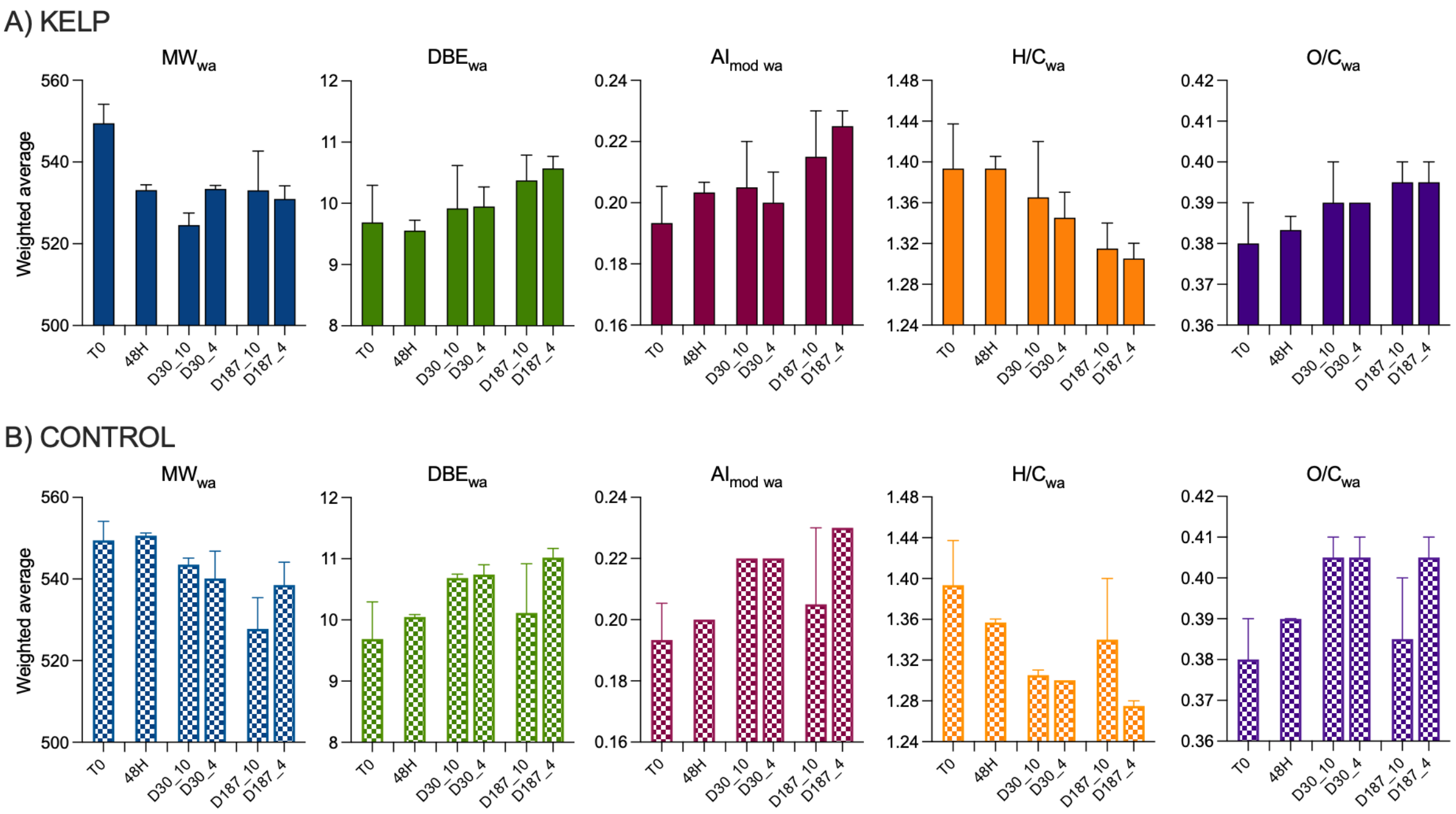

Figure S4.2: Weighted averages (wa) with standard error of means (SEM) for DOM metrics in kelp (A) and control (B) treatments during the production and biodegradation experiments, including molecular weight in g/mol (MW_{wa}), the double bond equivalent index (DBE_{wa}), the modified aromaticity index ($AI_{mod\ wa}$), hydrogen-to-carbon ratio (H/C_{wa}) and oxygen-to-carbon ratio (O/C_{wa}).

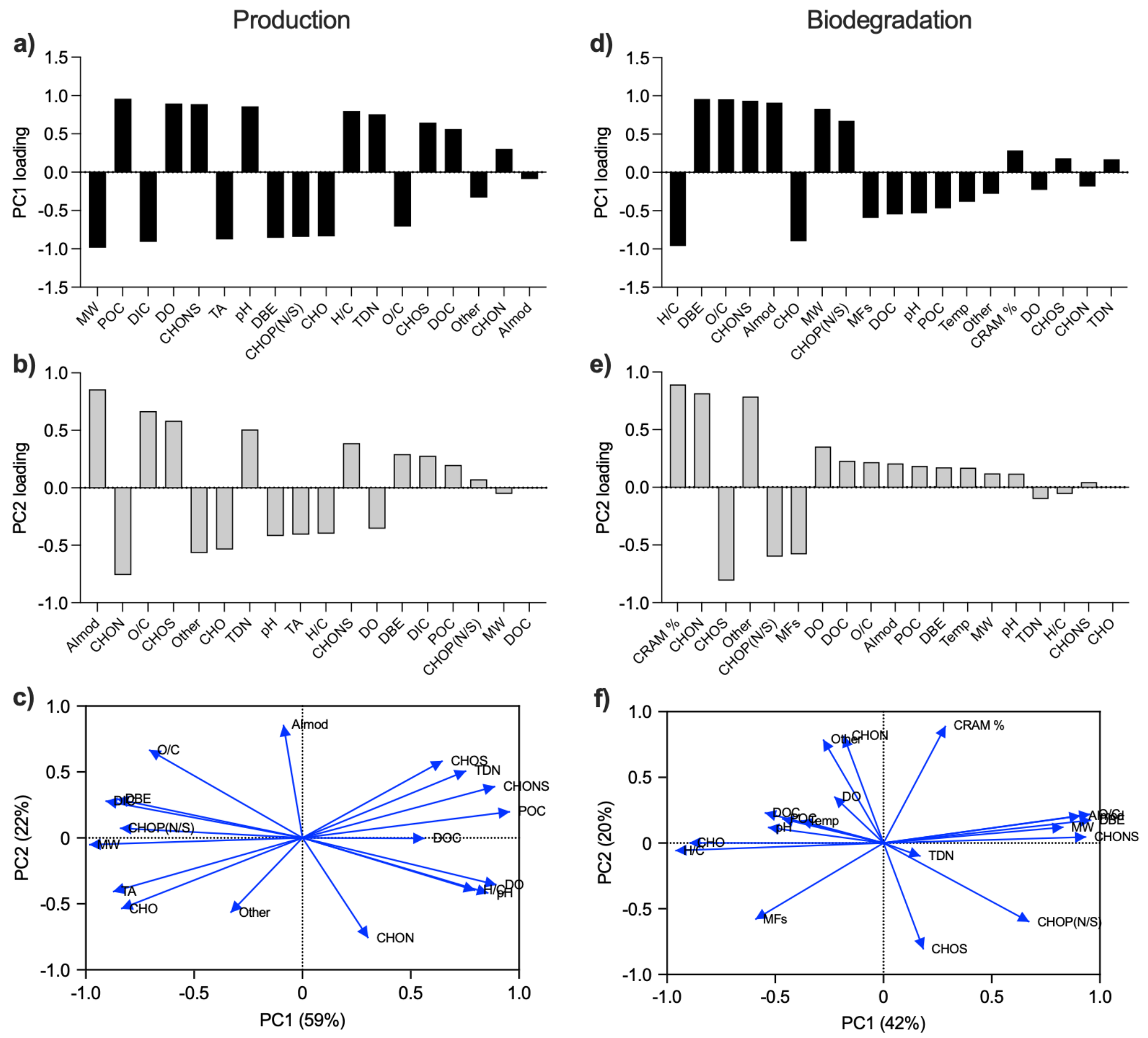

Figure S4.3: Loadings for principal components (PC) 1 and 2 for production (a-c) and biodegradation (d-f) experiments.

SI.5. Bacterial composition and diversity

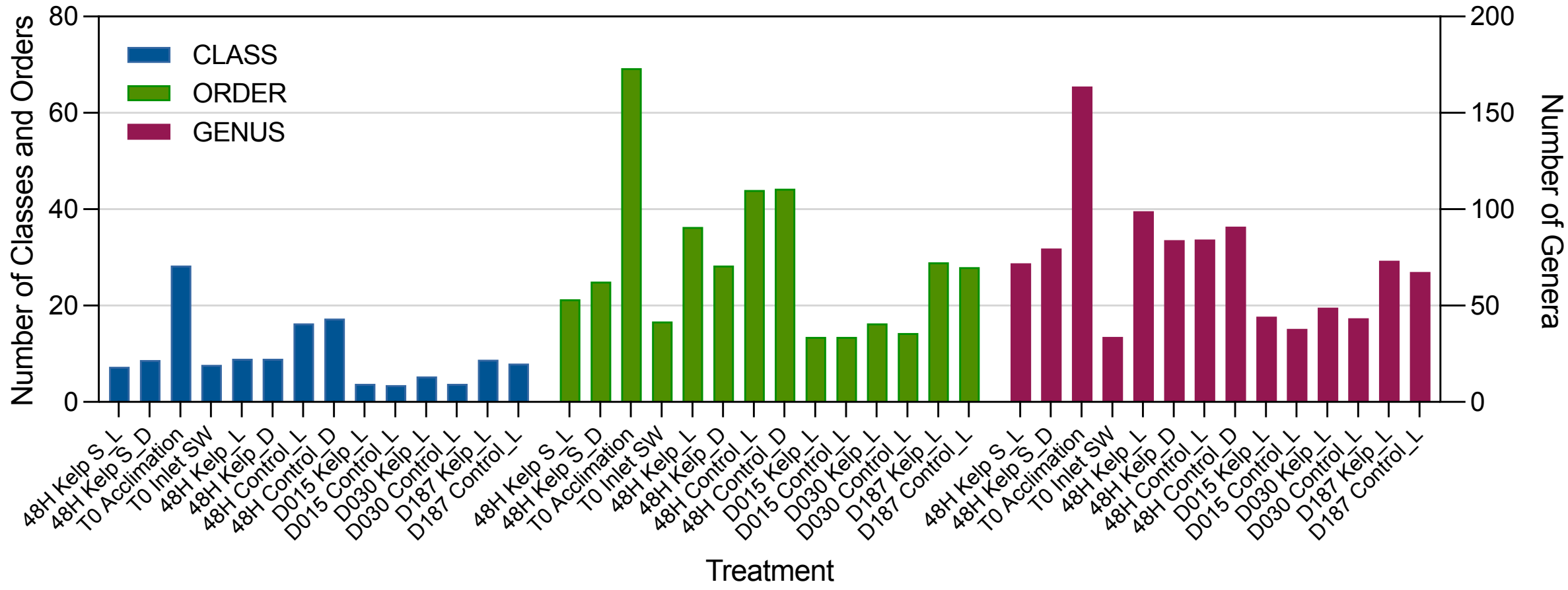

Figure S5.1: Summary of bacterial communities at class, order and genus level for all treatments during the production (T0 and 48H) and biodegradation experiments (D015, D030, D187). The production experiment occurred while exposed to daylight and biodegradation in darkness.

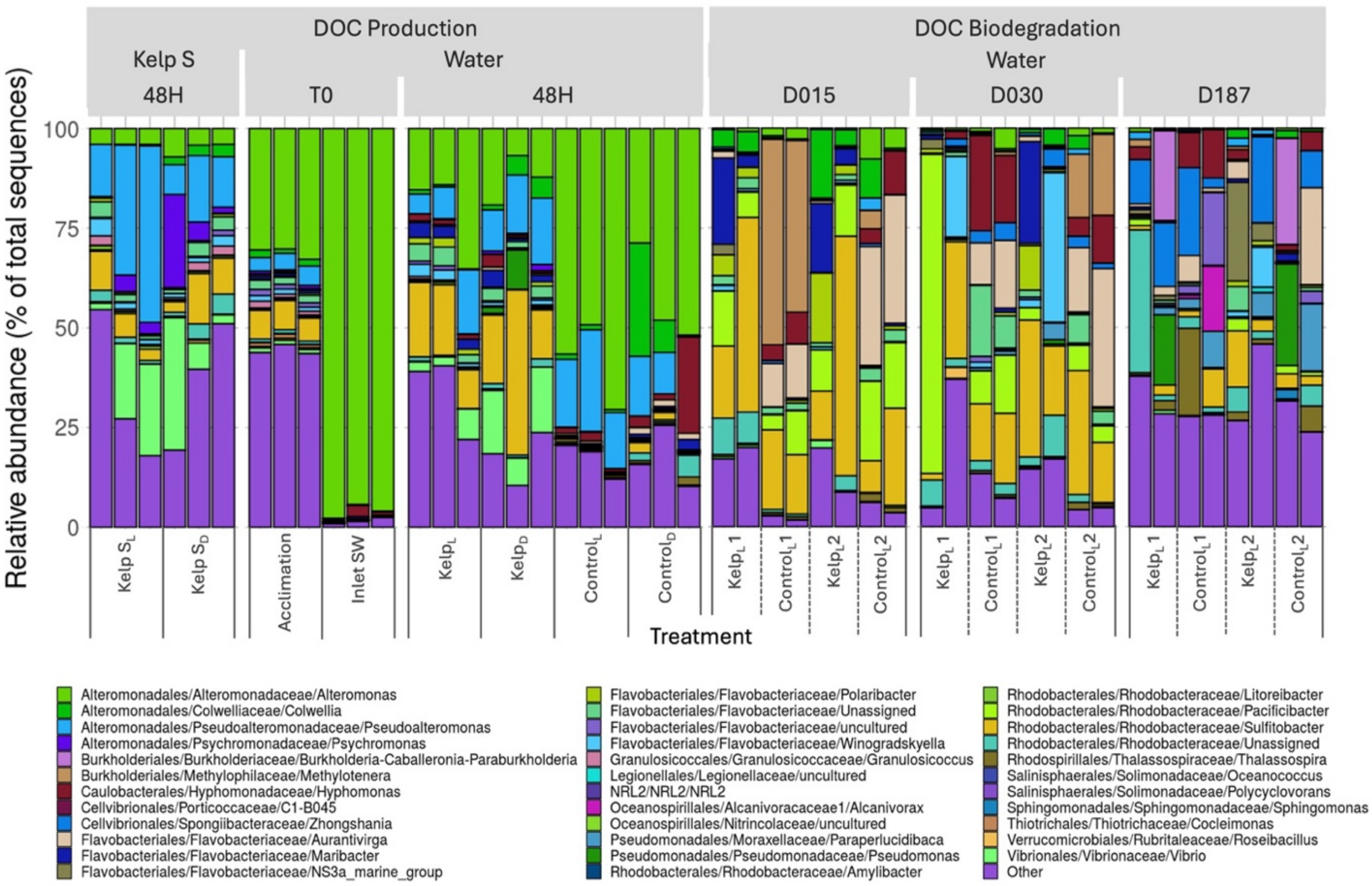

Figure S5.2: Bacterial community composition in kelp and water samples from production and biodegradation experiments to Genus level. Legends are shown as Order/Family/Genus. The production experiment occurred while exposed to daylight and biodegradation in darkness. Sampling done in duplicates (1 and 2) during the biodegradation experiment. Any taxa not reaching a relative abundance of 7% across all samples is grouped into "Other".

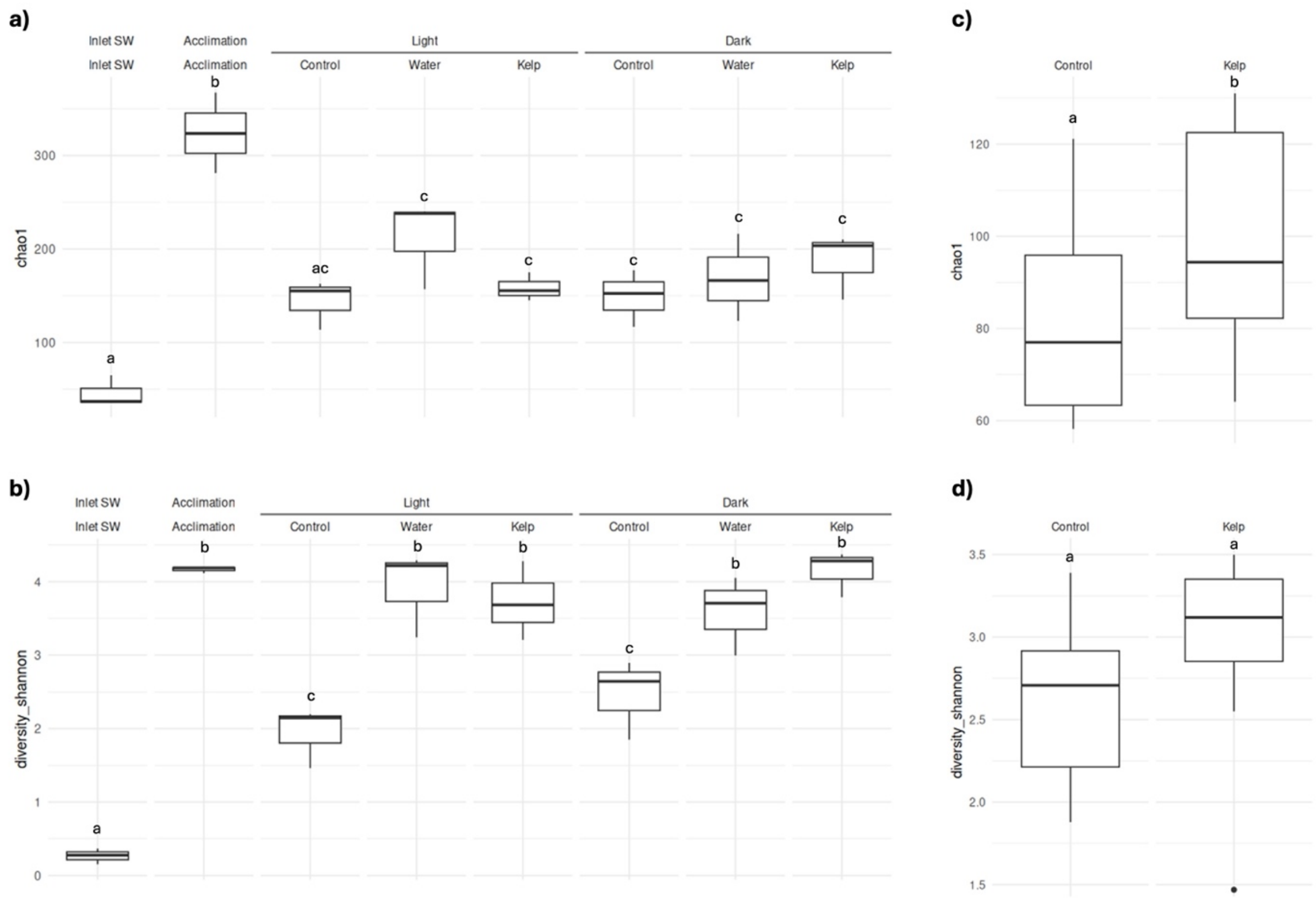

Figure S5.3: *Chao1* and *Shannon* diversity indices for the production (a-b) and biodegradation experiments (c-d). Kruskal-Wallis test was run for chao1 in biodegradation experiments as one of the requirements for data distribution was not met; 1. No outliers, 2. Normal data distribution, 3. Homogeneity. For all other ANOVA with post-hoc Tukey test was used. Letters inside panels indicate statistically significant differences.
